# Muver, a computational framework for accurately calling accumulated mutations

**DOI:** 10.1186/s12864-018-4753-3

**Published:** 2018-05-09

**Authors:** Adam B. Burkholder, Scott A. Lujan, Christopher A. Lavender, Sara A. Grimm, Thomas A. Kunkel, David C. Fargo

**Affiliations:** 1 Integrative Bioinformatics, National Institute of Environmental Health Sciences, NIH, DHHS, Research Triangle Park, Durham, NC 27709 USA; 2 Laboratory of Genomic Integrity and Structural Biology, National Institute of Environmental Health Sciences, NIH, DHHS, Research Triangle Park, Durham, NC 27709 USA

**Keywords:** DNA-seq, Indel, Mutation, Mutation accumulation, Mutation rate

## Abstract

**Background:**

Identification of mutations from next-generation sequencing data typically requires a balance between sensitivity and accuracy. This is particularly true of DNA insertions and deletions (indels), that can impart significant phenotypic consequences on cells but are harder to call than substitution mutations from whole genome mutation accumulation experiments. To overcome these difficulties, we present muver, a computational framework that integrates established bioinformatics tools with novel analytical methods to generate mutation calls with the extremely low false positive rates and high sensitivity required for accurate mutation rate determination and comparison.

**Results:**

Muver uses statistical comparison of ancestral and descendant allelic frequencies to identify variant loci and assigns genotypes with models that include per-sample assessments of sequencing errors by mutation type and repeat context. Muver identifies maximally parsimonious mutation pathways that connect these genotypes, differentiating potential allelic conversion events and delineating ambiguities in mutation location, type, and size. Benchmarking with a human gold standard father-son pair demonstrates muver’s sensitivity and low false positive rates. In DNA mismatch repair (MMR) deficient *Saccharomyces cerevisiae*, muver detects multi-base deletions in homopolymers longer than the replicative polymerase footprint at rates greater than predicted for sequential single-base deletions, implying a novel multi-repeat-unit slippage mechanism.

**Conclusions:**

Benchmarking results demonstrate the high accuracy and sensitivity achieved with muver, particularly for indels, relative to available tools. Applied to an MMR-deficient *Saccharomyces cerevisiae* system, muver mutation calls facilitate mechanistic insights into DNA replication fidelity.

**Electronic supplementary material:**

The online version of this article (10.1186/s12864-018-4753-3) contains supplementary material, which is available to authorized users.

## Background

Base substitutions in protein coding genes can result in detrimental codon changes, particularly with the introduction of a premature stop codon. Insertions and deletions (indels) can potentially cause frameshifts that change the translated sequence of the associated protein entirely. Large indels may result in complete loss of genes and key genetic control elements. Indels can change important spacing between genomic landmarks impacting trans-factor regulation. Though the impact of base substitutions is significant, the deleterious potential of indels is more substantial. Many studies have provided important insights into the mechanisms of indel generation (reviewed in [1]), including slippage of a DNA strand during replication of repetitive sequences [2]. The rate of indel generation during replication depends on the initial probability of slippage, the efficiency of exonucleolytic proofreading, and the efficiency of indel repair by the mismatch repair (MMR) machinery [3]. Indels in repeat tracts are diagnostic for disease. MMR defects, which cause rampant mutagenesis, genome instability, and cancer, result in characteristic microsatellite instability where indels expand or contract genomic repeat tracts. Defects in processes like genomic ribonucleotide excision repair (RER; [4–7], reviewed [8]), exonucleolytic proofreading [9, 10] and nucleotide homeostasis [11–13] are also characterized by indels in different repeat contexts.

Decades of in vivo mechanistic insights into these processes came from mutation rates derived from reporter gene assays [14]. When mutated, these reporter genes yield observable phenotypes. After accounting for silent mutations, penetrance, and expressivity, the frequency of phenotypic change may be related to the mutation rate. Given that rates of base-base substitution mutations tend to correlate across reporter genes, it is tempting to extrapolate reporter mutation rates to the genome scale; however, absolute rate estimates vary significantly between genes [15] and between genomic positions [16, 17], and no reasonable collection of reporter genes can capture the sequence diversity of a nuclear genome. In particular, long repeat tracts are rare in exons of protein coding genes [18], leading to underestimation of whole genome indel rates.

Whole genome mutation accumulation studies are an increasingly popular way to determine whole-genome mutation rates that addresses some shortcomings of reporter assays. This approach has been applied to eukaryotes as diverse as slime molds, ciliates, green algae, vascular plants, nematodes, arthropods, and vertebrates [9, 13, 18–48], including humans, if parent-offspring trios are included [49–53]. Mutation accumulation experiments have two characteristics that can improve discrimination and add sensitivity. First, mutation accumulation lines provide simultaneous access to both ancestor and progeny. Each sample may differ from the reference, but only variant alleles that also differ between ancestor and progeny are considered mutations. Unless truly gross changes occur during accumulation, progeny and ancestor should be largely isogenic and thus subject to the same sources of mapping and sequencing error at each locus. Second, careful controls on selective pressures mean that no sub-population can grow to an unrepresentative fraction of the total population. Thus, allelic fractions in the final progeny sample will be constrained by those in the last founder cell, simplifying construction of genotype models. Tools that leverage aspects of mutation accumulation experiments to call substitutions with extremely low false positive rates are an active area of research [44], however those currently available do not identify insertions or deletions.

Mutation accumulation experiments ease modeling and discrimination of mutations but there are further challenges to mutation detection. Errors due to library preparation, sequencing biases, and mismapping make indels harder to detect and characterize than substitution mutations [54, 55]. In addition, repeat tracts increase sequencing errors due to amplification [56, 57] and mapping errors due to misalignment, especially when repeat tracts approach or exceed read length (reviewed [58]). We recently used whole genome mutation accumulation experiments to probe both substitution and indel rates in diploid *Saccharomyces cerevisiae* strains that encoded either wild type or mutator variants of DNA Polymerases α, δ or ϵ (Pols α, δ or ϵ) and were either proficient or deficient in MMR [18]. The > 35,000 base substitutions observed were sufficient to characterize the division of labor between replicative polymerases, to describe the efficiency of base-base MMR, and to differentiate rates with respect to genomic landmarks. False positive mutation calls are particularly problematic when the underlying mutation rate is low. Since all samples were compared to *wild type* cells with very low mutation rates, stringent mutation calling filters were used to minimize the false positive rate (FPR). However, replicate sequencing libraries suggested that about a quarter of substitutions from any given library were not called, and statistical projections suggested that indel sensitivity was even lower [9]. Low sensitivity is problematic if statistical testing is underpowered with a low number of mutations or if sensitivity varies with context, as we saw in repeat tracts of varying length. To correct for such underestimation, a statistical correction was created to extrapolate from high-confidence conservative indel calls based on assumptions about the primary sources of error. This revealed some mechanisms and consequences of proofreading defects [9] and altered nucleotide pools [13], but was of limited utility where indel and/or repeat tract counts were low and stochastic effects were inflated by the extrapolation.

Here we introduce muver (*mutationes verificatae*), a mutation calling framework that streamlines the use of common tools and applies novel methods to leverage the power of matched progeny and ancestor to minimize the FPR without sacrificing sensitivity. Muver includes detailed analyses of underlying mapping patterns, possible mutational pathways, and explicit recognition of ambiguity to increase both the accuracy and sensitivity of mutation calling. This combination of features is necessary for the accurate estimation of mutation rates. Though designed to improve mapping of indels in repeats, muver also improves substitution calls with published data [18]. Muver results compare favorably to previous statistical projections [9] without increasing calls in control samples, suggesting low false negative and false positive rates. Muver reanalysis extracts additional detail, meaning, and mechanistic insight into MMR and DNA replication fidelity.

## Methods

### Strains, mutation rates and analysis of *URA3* mutants

Measurements of spontaneous mutation rates and the sequencing of *URA3* mutants were as previously described [59–61]. *URA3* rates used here are from two previous studies [62, 63]. *Saccharomyces cerevisiae* strains, strain construction, mutation accumulation experiments, genomic DNA preparation, Illumina library preparation, genome sequencing, reference genome assembly, and genomic feature selection proceeded as previously described [18]. Strains were diploids descended from Δ|(− 2)|-7B-YUNI300 (Pavlov et al. 2001) and were homozygous for *CAN1*, *his7-2*, *leu2-Δ*∷*kanMX*, *ura3-Δ*∷, *trp1-289*, *ade2-1*, and *lys2-*Δ*GG2899-2900*.

### Mutation accumulation experiments

Descendant samples are compared to ancestral samples to specifically detect mutations that accumulate over the course of the experiment and to exclude changes that occur during strain construction and handling. Bottlenecks may be introduced to fix existing mutations; after the bottleneck, all descendants will share a mutation set, and none will have a selective advantage. As in previous studies [18, 24], bottlenecks were introduced through streaking on solid media. Samples were identified by passage number, with the ancestor sample defined as t0 (“time point 0”). The whole genome mutation accumulation data presented here was collected previously [18]. Raw data resides at the National Center for Biotechnology Information (NCBI) Gene Expression Omnibus under accession number GSE56939.

### The muver framework

Muver is a packaged Python framework written and tested using Python 2.7.13. All muver functions can be accessed using its command-line interface. Muver is open-source (MIT license), with code available in a public online repository (https://github.com/niehs/muver). Thorough documentation detailing instructions for installation and usage is included.

**Fig. 1:**
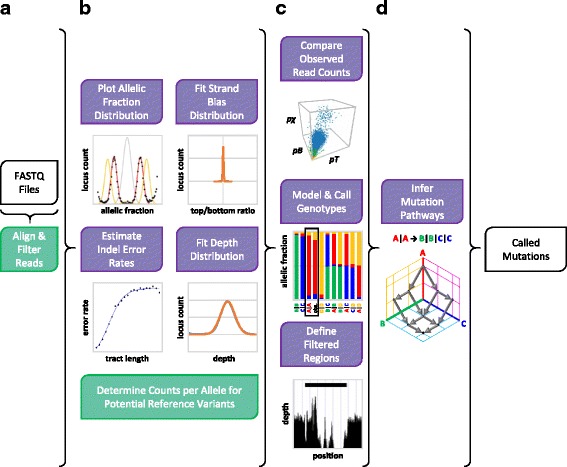
Overview of muver operation. White boxes represent input/output files, purple boxes represent tasks implemented in the muver codebase, while green boxes represent tasks performed by external tools. **a** Given a list of FASTQ files, muver initiates alignment, quality filtering, and deduplication of reads. **b** For each sample, Gaussian distributions of per-nucleotide coverage and bias in coverage per-strand are fit to observed values to aid in filtering of sites with abnormal coverage patterns, and indel error rates are estimated as a function of repeat length. Optionally, allelic fraction distributions are generated to facilitate determination of sample ploidy. Read counts supporting each observed allele, per-strand, are determined for potential reference variants using GATK HaplotypeCaller. **c** Outgrowth (descendant) sample read counts are compared to t0 (ancestor) using three separate statistical tests; genotypes are called for each sample by comparing observed read count distributions to those expected for all possible combinations of observed alleles at the provided ploidy. Regions with abnormally high or low coverage, to be excluded from mutation calling, are identified based on the previous fit. **d** For each outgrowth sample, mutations are inferred at sites where read counts differ sufficiently from t0 by enumerating all possible series of events that explain the called t0 and outgrowth genotypes. The most parsimonious of these are accepted as the most likely, and events occurring in all cases are reported to the output

Prior to analysis, supporting reference files are generated using the “index_reference” and “create_repeat_file” commands (see “Reference genome preparation”; Additional file 1: Figure S1). This step must only be completed once per assembly. Remaining tasks are performed by the “run_pipeline” command. Analysis requires one or more outgrowths with a single t0 control sample, as such “run_pipeline” requires a text file describing attributes of each sample in the analysis, including paths to associated FASTQ files and sample ploidy. This step performs the alignment and filtering of reads (Fig. 1a), observes patterns of sequencing depth, strand bias, and indel errors (Fig. 1b), and finally performs genotype calling and identification of high-confidence mutations (Fig. 1c-d). These steps are detailed below. Output files are generated that describe the observed depth distributions, strand bias distributions, and estimated indel error rates in plain text format. Read coverage for each sample is output in bedGraph format, along with filtered regions in BED format; mutation calls are written to a tab-delimited text file, as well as a file in VCF format.

Muver was designed to allow iterative refinement of mutation calls. For instance, the sequencing depth may be examined for bias using the “calculate_depth_ratios” command. If necessary, depth correction may be performed, and observation of genome-wide sequencing depth re-run using the “correct_depths” and “calculate_depth_distribution” commands. Mutation calling can then be performed again based on updated depth observations using the “call_mutations” command. Similarly, the “plot_allelic_fraction” command may be utilized to assess global ploidy, and mutations can be re-called under revised assumptions of whole-genome copy number.

The muver framework calls established bioinformatics tools to perform common read processing and alignment functions and to determine observed read counts at potential variant sites. Read processing is performed by samtools version 1.3.1 [64] and Picard version 2.9.2 (http://broadinstitute.github.io/picard/). Alignment is performed by Bowtie 2 (version 2.3.0) [65]. Local reassembly of reads and determination of counts supporting all observed alleles is performed by the Genome Analysis Tool Kit, or GATK (version 3.7-0) [66].

### Reference genome preparation

Prior to analyzing sample data with muver, indices and other files must be generated from the reference genome sequence. These include a Bowtie 2 index, constructed using bowtie2-build with default parameters, a FASTA dictionary file, generated using Picard CreateSequenceDictionary, and a FASTA index file, generated using samtools faidx. In addition, a list of all simple repeats with unit length 1-4 and total length 4 or greater is identified, eliminating those that either could be reconstructed through concatenation of shorter units or that are wholly contained within another tract of the same repeat unit or a circular permutation thereof. Optionally, “extract_repeat_file_sample” can be utilized to draw a smaller random sample of repeats from the full list. This file is utilized by “run_pipeline” automatically during the assessment of indel rates described below, and reduces the memory required for the analysis of large genomes.

### Alignment and identification of variants

Potential variants from the reference in all samples are identified using GATK’s HaplotypeCaller. Earlier iterations of this pipeline utilized the called genotypes produced at this stage, however, we found many cases where heterozygous genotypes were called with relatively low allelic fractions, particularly within repeats. Most of these appeared to be the result of PCR errors accumulated during library preparation and sequencing, while a minority were likely the result of true mutations occurring within the first few cell divisions after the final passage. As the largest effect of this phenomenon occurs within simple repeats, particularly homopolymers, muver seeks to observe and account for this on a per-sample basis. Application of GATK’s Base Quality Score Recalibration and tuning of other parameters reduced the incidence of these miscalls, but did not eliminate them entirely. For this reason, we elected to perform our own genotype calling, still utilizing HaplotypeCaller to identify sites of interest and taking advantage of its local reassembly of reads to reduce errors in regions where the initial local alignment performed poorly. GATK’s documentation states that the counts returned by the StrandAlleleCountsBySample annotation may include reads are filtered during genotype calling. Due to our pre-filtering of reads with low alignment scores, and the fact that we do not attempt to interpret the read counts directly as allelic fractions, we believe that these values are a suitably reliable source of count information.

Reads are aligned using Bowtie 2’s sensitive local alignment mode to maximize the possibility of observing indels, allowing fragment lengths of 0 to 1000 bp by default (−-fr --local --sensitive-local -I 0 -X 1000). Pairs with reads mapping to distinct chromosomes are then filtered due to a high likelihood of misalignment, as well as reads with alignment quality less than 20. Read group tags are added to the resulting SAM file to allow compatibility with GATK, and duplicate fragments are filtered using Picard MarkDuplicates (REMOVE_DUPLICATES = TRUE), generating an output file in BAM format. Following creation of the required BAM index file with samtools, local realignment is performed using GATK’s RealignerTargetCreator and IndelRealigner modules. Due to the expectation of high-depth data sets, the maximum allowed read count in an interval considered for realignment was increased to 100,000 (−-maxReadsForRealignment 100,000). Picard FixMateInformation module is then utilized to ensure all BAM records accurately reflect the changes to the alignment implemented in the previous step. Potential variants from the reference in all samples are then identified using GATK’s HaplotypeCaller using default parameters, with the following exceptions: the minPruning and minDanglingBranchLength arguments, which affect the number of haplotypes retained in the assembly step, are set to 0 to maximize sensitivity, and counts of supporting reads per sample, for each allele and on each strand are requested (−-minPruning 0 --minDanglingBranchLength 0 -A StrandAlleleCountsBySample).

### Observation of genome-wide depth of coverage and strand bias

Two common causes of incorrect genotype, and therefore false positive mutation calls, are genomic regions where reads map poorly and regions of abnormal depth, each resulting in skewed allelic fractions. In both cases, greater variation in read coverage is observed than would be expected by chance. Observation of the frequency of averaged depths across a very large number of samples revealed these distributions to be approximately normal. Muver assesses depth of coverage genome-wide for all samples, and these distributions are used to locate regions of abnormally high or low coverage. Most of these result from alignment deficiencies, but some may result from true copy number variations (CNVs). Muver allows an optional input of per-position ploidy values, which may be found using a CNV caller. If such a file is provided, muver will make genotype calls considering ploidy at each position.

Another source of incorrect genotypes, affecting primarily subclonal genotype calls, is the mismapping of reads in regions on the periphery of unique and non-unique genomic sequence. These reads typically represent a minority of the total count, and generally align to a single strand. These features allow such miscalls to be detected through assessment of allele strand bias. Observations of natural log-transformed strand bias values were found to be approximately normal. In cases where a subclonal genotype is called, these distributions are utilized to detect abnormal strand bias in the differentiating subclonal allele, allowing reversion to the primary clonal genotype where such bias is present.

To characterize genome-wide read coverage the samtools mpileup function is used, disabling its probabilistic realignment (BAQ), applying the same mapping quality and base quality thresholds used by HaplotypeCaller (20 and 10, respectively), and setting an arbitrarily large maximum read count per position, 100,000, to ensure all reads are considered (-B -Q 20 -q 10 -d 100,000).

The mpileup output is used to determine regions of the genome with abnormal depth that are subsequently filtered. First, a Gaussian distribution is fit to observed read depths, scaled by the user-provided ploidy or local copy number. Then per-position read depths are averaged using a 25-bp moving window. Average values are then compared against the cumulative distribution function of the fitted distribution, generating a two-tailed *p*-value. Positions with *p*-values below a given threshold (default = 1e-4), falling within a given maximum distance (default = 1000), define the boundaries of regions to be filtered, provided their depths both fall above or below the mean. Intervals defined for each sample are written to bed format files supporting subsequent inspection.

The mpileup output is also used to characterize strand bias in each sample. For each position, the ratio of the top strand counts to the bottom strand counts is found. A Gaussian distribution is fit to the observed frequencies of the natural log-transformed ratios. Distribution parameters are retained to assess strand bias during mutation calling.

### Correction of bias in depth of coverage

Muver is capable of depth correction for systemic mapping biases in sequencing depth and library construction artifacts. Visual inspection of the bedGraph files produced while mapping genome-wide coverage revealed that depth was often highest at chromosome ends. While the cause of this issue is unknown, we found that the magnitude of the effect scaled regularly with distance from the nearest chromosome end, and could be reliably modeled by the sum of a log-normal cumulative distribution function and linear function:

$$ factor= scalar\left(1-\left[\ \frac{1}{2}+\frac{1}{2}\operatorname{erf}\left(\frac{\ln x-\mu }{\sqrt{2}\ \sigma}\right)\right]\right)+ intercept+ slope\ x $$

where *x* is distance from the nearest chromosome end in bps and *factor* is the expected depth at that position relative to the genome-wide mean. To facilitate estimation of the parameters *intercept*, *scalar*, *μ*, *σ*, and *slope*, the ratio of coverage at each position relative to the genomic mean is determined and placed in 500-bp bins based on distance from the nearest chromosome end. The median for each bin is then determined, after excluding extreme values above 4 or below 0.25. Parameters from the equation above can be estimated from these ratios by performing a non-linear least squares fit within R, Matlab, Excel, or another preferred tool. Following fitting, depth values may be corrected by passing distribution parameters to the muver function “correct_depth”, where observed depth values are scaled by $\frac{1}{factor}$. Regions of abnormal coverage and strand balance may then be identified using the functions “calculate_depth_distribution” and “calculate_bias_distribution”.

This module is optional and may be replaced by other systemic depth correction tools.

### Assessment of per-sample indel error rates

Insertions and deletions in repeat sequences are introduced by PCR during library preparation and sequencing and are a major source of error during genotype calling. Muver corrects for these errors based on the assumption that most insertions and deletions in repeats are attributable to these sources. We find the observed rates vary across event types (insertion or deletion), lengths of repeat units, and lengths of the overall repeat tract. To correct for these errors, muver counts reads containing an indel that fully traverse a previously identified repeat tract. For each sample, these values are summed for each combination of unit length (1-4 nt) and total tract length and expressed as a ratio of observed insertions and deletion to overall coverage. We consider these ratios as the estimated insertion and deletion error rates in repeat regions. Muver fits the observed rates to the following function based on repeat tract length:

$$ error=M+\frac{L}{1+{e}^{-k\left(x-x0\right)}} $$

Here, *x* is repeat tract length, and M, L, k, and × 0 are parameters that describe the fit. For each sample, fits are performed separately for insertions and deletions and for unit lengths of 1, 2, 3, and 4 nucleotides. Rates were calculated based on fewer than ten distinct repeat loci are not considered during fitting. The fitted parameters *M*, *L*, *k*, and *× 0* are retained to determined expected insertion and deletion rates during genotype calling.

### Estimation of global ploidy

Muver considers the ploidy of each sample during genotype and mutation calling. Muver facilitates estimation of ploidy by providing a distribution of observed allelic fractions for each sample. To this end, read counts supporting each allele at all positions genome-wide are determined using samtools mpileup (-B -Q 20 -q 10 -d 100,000). Allelic fractions are calculated as the ratio of reads mapping to a given allele to the total read count at a given position. A histogram of observed allelic fractions is then calculated and reported. In most cases, the rate of heterozygosity is low, and these distributions display very high counts close to zero and one, although, minor peaks are usually apparent, and their number and location provide an indication of ploidy. The largest of these peaks should occur at $\frac{1}{ploidy}$ and, for ploidies greater than 2, $1-\left(\frac{1}{ploidy}\right)$ (Additional file 1: Figure S2).

### Genotype calling

Genotypes for each outgrowth and control t0 sample are called by selecting the genotype whose expected allelic frequencies most closely match observed frequencies. We assume that the observed allelic frequencies are due to contributions from a clonal genotype and a potential sub-clonal genotype at a given sub-clonal frequency. To call, first all possible clonal and sub-clonal genotypes are enumerated considering sample ploidy and observed alleles, and the expected allelic frequencies are determined. We allow sub-clonal genotypes to be only one allele different than the clonal genotype. We allow sub-clonal allele frequencies to be 0.500, 0.250, or 0.125, corresponding to mutations occurring in the first, second, or third generations. For a clonal genotype, sub-clonal allele, and sub-clonal frequency, the expected frequency for an allele is calculated by the following:

$$ freq.=\left(1- subclonal\ freq.\right)\times \frac{allele\ count, clonal\ }{total\ clonal\ alleles}+ subclonal\ freq.\times \frac{allele\ count, subclonal}{total\ subclonal\ alleles} $$

Anticipating elevated PCR error in repeat sequences, the expected allelic frequency is corrected based upon the previously observed error rate. For each allele, the insertion and deletion error rates are calculated based on the unit length and the total repeat tract length. We find that the insertion or deletion of a single unit is by far the most commonly observed type of error. As such, correction factors are applied to alleles that result from the insertion or deletion of single unit. For example, given a genotype of AA|AA, and expected + 1 and − 1 unit error rates of 0.03 and 0.05 respectively, the corrected excepted frequencies would be as follows: A - > 0.05, AA - > 0.92, AAA - > 0.03. Following error correction, all frequencies of zero are instead set to $\frac{2}{total\ sample\ depth}$, which corresponds to a pseudo-count added for each strand and prevents division-by-zero-errors in subsequent scoring.

Following calculation of expected frequencies, observed allele frequencies are calculated considering per-allele and total read counts for each sample. Each potential genotype is scored using the following for expected rates *e*_*0*_ … *e*_*n*_ and observed rates *o*_*0*_ … *o*_*n*_ corresponding to alleles *a*_*0*_ … *a*_*n*:_

$$ score={\sum}_{i=0}^N abs\left[\mathit{\ln}\left(\frac{o_i}{e_i}\right)\right] $$

The single genotype with the minimum score is accepted as the called genotype. For ties when calling the t0 genotype, the genotype containing the most occurrences of the reference allele is selected. For outgrowth samples, the genotype containing the highest number of alleles shared with the t0 call is selected.

If the called genotype contains a subclonal element, two additional statistical tests are performed. First, the observed rate of reads attributed to the differentiating subclonal allele is compared to the rate that would be expected given the clonal genotype call by binomial test. An upper-tail *p*-value is derived from the cumulative distribution function, and compared to the threshold that controls family-wise error rate (FWER) at a user-specified rate (default = 0.01) using Šidák correction (19) with the number of positions in the genome considered as the number of tests performed. If the *p*-value exceeds the threshold, only the clonal genotype is reported. Second, the strand bias of the reads attributed to the subclonal allele is examined to ensure that the call is not an effect of mismapping. The natural-log transformed ratio of top strand to bottom strand counts is determined, and a two-tailed p-value is calculated against the genome-wide distribution. The subclonal call is not retained if the p-value falls below the FWER-based threshold. If abnormal strand bias is detected in a called subclonal allele in a t0 sample, no mutations are reported at that position, due to the high likelihood of erroneous calls. Additional sources of error may be present that manifest as apparent subclonal mutations, that are not accounted for in muver’s analysis. As such, reported subclonal calls should be carefully scrutinized (see Additional file 1: Results and Discussion).

### Identification of mutations

Mutations are identified through comparison of the read counts per strand and allele reported by HaplotypeCaller for a given t0 and outgrowth sample pair. The comparison is not performed at positions where counts are observed for either sample on a single strand only or for a single allele (e.g. 25 reads supporting allele “A” mapped to the minus strand only). This scenario is commonly associated with mismapping of reads on the periphery of regions with non-unique sequence. In addition, for the data sets presented herein, we have excluded regions where we have historically observed poor read coverage: telomeres; sub-telomeres; rDNA loci; and small regions flanking each of these.

The development of muver was aided by a test data set with approximately 21,000 potential mutations identified with GATK HaplotypeCaller set to maximize sensitivity (Additional file 1: Figure S3). Initially, read counts were compared separately per-allele and *p*-values were determined from the upper tail of a binomial distribution. Sites were rejected for p-values above a threshold chosen to control the family-wise error rate (FWER) at 0.01 via Šidák correction (68), assuming a number of tests equal to the count of queryable (i.e. not excluded) positions in the genome. Upon implementation, false positives were noted, which upon further inspection, appeared largely due to an increase of reads, likely mismapped, attributed to only one strand. This issue was resolved by performing the binomial tests separately for each strand and requiring that *p*-values of both fall below the threshold (Additional file 1: Figure S3A; “auto positive,” in blue). While this avoided most manually classified false positives (Additional file 1: Figure S3A; “manual negative,” in orange), a large number of manually classified false negatives were observed (Additional file 1: Figure S3A; “manual positive,” in green). A third test, the chi-square goodness of fit, was implemented to compare the full distributions of counts across all alleles and strands. When this test metric was compared to either binomial *p*-value, the “manual positive” and “manual negative” data clouds appear to interpenetrate to a high degree (Additional file 1: Figure S3B). In three dimensions, with each test on a separate axis, nearly all false positive mutations fall close to one of the axes or very near the origin (Additional file 1: Figure S3C). Based on these observations, muver rejects any variant site with one or more *p*-values greater than 0.1, as well as any site for which the vector sum of the transformed *p*-values was less than the FWER-corrected threshold.

Therefore, muver makes mutation calls considering multiple statistical tests:

- A chi-square goodness of fit test is used to compare read counts of the outgrowth to the control t0 sample. The test considers read counts at each allele and each strand separately, resulting in the *p*-value p_chi_.
- An upper-tail p-value is generated by comparing the outgrowth allelic frequency to the binomial cumulative distribution function defined by the t0 allelic frequency. A pseudo-count is added where the read count is 0. Each strand is considered separately, yielding p-values p_top_ and p_bottom_.

The computed p-values from both chi-square and binomial tests are used to call mutations. For each allele, a composite score is calculated as follows:

$$ score=\sqrt{{\left({-\mathit{\log}}_{10}{p}_{top}\right)}^2+{\left({-\mathit{\log}}_{10}{p}_{bottom}\right)}^2+{\left({-\mathit{\log}}_{10}{p}_{chi}\right)}^2} $$

This score represents the distance from certainty that no mutation occurred, and is considered against the similarly log-transformed p-value threshold necessary to control the FWER at a user-specifiec rate (default = 0.01, Additional file 1: Figure S3). To be called as a position with a putative mutation, p_chi_ must be less than 0.1, and there must exist at least one allele where the composite score is greater than the FWER-derived threshold and both p_top_ and p_bottom_ are less than 0.1.

### Inference of mutation identity

For called mutations, individual mutation events are identified based on genotypes called in the outgrowth and t0 control. To do this, all possible mutation events are determined given observed alleles. The considered mutations are as follows:

- Conversion of one allele to another (substitution, insertion, or deletion)
- Gain of one allele (copy number gain)
- Loss of one allele (copy number loss)

Considering the t0 genotype as a starting point, sequences of mutation events (or paths) are considered iteratively until one or more sequences of mutation events are found that results in the outgrowth allele. If more than one path is found, only mutation events shared across all sequences are reported (Additional file 1: Figure S4).

Muver captures mutation events that are ambiguous but still informative. In a second pass, mutations present in all sequences that are of the same type (conversion, gain, or loss) and that share the same starting allele are grouped together and reported as ambiguous. These ambiguous events can be important for subsequent analysis. For instance, these ambiguous events can result from CNVs when the identity of the gained or lost allele is ambiguous.

Clusters of mutations that increase the count of an existing allele but do not increase copy number can point to large scale structural rearrangements or gene conversion events. To facilitate discovery, these events are flagged as potential allelic conversion events, or PACs. If a mutation is ambiguous, the PAC flag may be ambiguous as well, depending on its constitutive alleles. If so, the PAC flag is explicitly defined as ambiguous.

Mutations are reported in the format recommended by the Human Genome Variation Society (HGVS) and by NCBI. We also use the following rules to describe mutations for which the HGVS nomenclature was not designed, primarily CNV events and mutations to non-reference alleles:

- A copy number gain is reported using the keyword “gain”, e.g. g.1000gainA.
- A copy number loss is reported using the keyword “loss”, e.g. g.1000lossA
- A copy number variant with an ambiguous allele is reported with an asterisk, e.g. g.1000gain*.
- To report a mutation in a non-reference allele, the mutation is identified using the flanking nucleotides, e.g. for a G to C substitution at 1000A, the mutation would be reported as g.999_1001G > C.
- Ambiguous mutations are grouped with pipes, e.g. g.1000A > C|g.1000A > G.

### Filtering and reporting of results

Mutation calls are filtered if any of the following are true:

- Muver allows regions excluded from analysis to be set by the user through an input BED file. All mutations in these regions are filtered.
- Read depths in the outgrowth or t0 control are differ significantly from the global mean.
- Read depths in the outgrowth or t0 control are below a depth threshold (default = 20 reads).
- Strand bias in the t0 control subclonal allele differs significantly from the global mean.
- If only one allele has coverage in the outgrowth or t0 control, and that allele only has coverage on one strand.
- Mutation calling score or individual *p*-values do not meet described thresholds.

Mutations that pass filters are reported in two output formats: a tab-delimited text file and a VCF file.

### Assessment of muver sensitivity and false positive rates with a human gold standard

High confidence variants relative to the hg19 reference genome called by the Genome in a Bottle Consortium [67] (release 3.3.2) for the father (NA24149) and son (NA24385) of the Ashkenazim Trio were used to compile a set of sites known to differ between individual samples, and a subset of the raw data was analyzed to measure muver’s sensitivity and false positive rate. For this analysis, we utilized the first 4,000,000 reads from each of the 336 and 288 HiSeq lanes generated for the HG003_HiSeq300x_fastq (father) and HG002_HiSeq300x_fastq (son) data sets, respectively. These 1.23 and 1.14 billion paired-end, 150 nt reads provided an estimated 126× and 108× coverage, reducing the depth to a level more readily achieved in experiments likely to be analyzed using muver. To provide context for these values, we identified clonal differences with VarScan version 2.4.3 [68] using the Somatic Mutation Calling workflow and VarDict version 1.5.1 [69]. Additionally, we called variants with HaplotypeCaller for the father and son individually, and combined the results to identify differences.

Muver analyses were performed using a sample of 5,000,000 repeat loci for the estimation of indel error rates. For the purposes of this analysis, the father was considered the “t0” sample, and the son the “outgrowth”. Similarly, within the VarScan analysis, the father was considered the “normal” sample, and the son the “tumor”. Analyses were performed with each tool across a range of values of the primary tunable parameter: a FWER of 2 × 10^− 7^ to 0.9 for muver, somatic *p*-value of 1 × 10^− 50^ to 0.1 for VarScan, a p-value of 1 × 10^− 6^ to 0.1 for VarDict, and a calling confidence threshold of 10 to 10,000 for HaplotypeCaller (Additional file 1: Tables S5-S8).

Input files for VarScan were generated from the BAM files produced during analysis with muver, using samtools mpileup, applying mapping and base quality filters of 20 and 10 and setting the maximum depth to an arbitrarily large value to ensure all reads are considered (−d 100,000). VarScan’s somatic function was run using default parameters. Results were filtered using its included processSomatic tool accepting mutations classified as “high confidence” and categorized as “Somatic” or “LOH”, as well as those labeled “Germline” where the called genotypes differed.

Analysis with VarDict was performed utilizing the tool’s paired sample mode with minimum allelic fraction set to 0.1, the same applied by VarScan, but otherwise default or author-recommended parameters. Calling was limited to the regions defined by the Genome in a Bottle Consortium as high confidence in both the father and son. To facilitate comparison with the Genome in a Bottle calls, complex mutations reported by VarDict were broken into their constituent substitutions and indels using *vcfallelicprimitives*, a component of vcflib v1.0.0-rc1.

Base quality score recalibration (BQSR) was performed prior to analysis with HaplotypeCaller using GATK’s BaseRecalibrator function, passing the dbSNP build 150 “common_all” vcf file as the required list of known sites, and considering all default covariates, as well as repeat unit and repeat length. HaplotypeCaller was run for each sample individually in GVCF mode (-ERC GVCF), and results were combined using GenotypeGVCFs.

Differences identified by any tool outside of the high confidence regions defined by the Genome in a Bottle Consortium in both the father and son data sets were not considered in the determination of false positive and false negative rates. Similarly, individual sites or regions automatically excluded by muver due to abnormal depth or coverage patterns were not considered, as these were determined individually per-sample, independent of any annotation or consideration of sequence content, by a central component of the analysis pipeline. Such exclusions were not possible for HaplotypeCaller, VarScan, or VarDict, which do not explicitly define high or low confidence regions in their respective outputs. In all cases the entirety of chromosomes X, Y, and the mitochondrial genome were also excluded.

### Assessment of muver performance within yeast mutation accumulation experiments

To further assess muver’s performance, we compared mutation calling results with VarScan as well as MuTect2 (beta) [70] in the context of *S. cerevisiae* mutation accumulation experiments. Input files for VarScan were prepared and results filtered as in the analysis of human data above. Base quality score recalibration was performed on alignment files prior to running MuTect2. To generate the necessary lists of sites with known variation, to be excluded during BQSR, the t0 samples of each data set were examined with HaplotypeCaller. Aside from requesting the StrandAlleleCountsBySample annotation, default parameters were used. A set of conservative filters were then applied to the variants, accepting only those whose allelic fractions were less than 0.1 from the value implied by the called genotype, and whose supporting reads did not display a strand bias greater than 0.9. These high confidence variants were passed to the BaseRecalibrator tool, which was run considering the default covariates as well as repeat unit and repeat length. MuTect2 was run using default parameters, passing outgrowths and t0 as matched tumor-normal pairs. MuTect2 results were filtered based on the following flags: t_lod_fstar, germline_risk, triallelic_site, homologous_mapping_event, multi_event_alt_allele_in_normal. Mutations flagged as “alt_allele_in_normal”, “clustered_events”, or “str_contraction” were accepted, as no such filters are applied by muver. An additional step was applied to the MuTect2 results: mutations were filtered whose outgrowth alternate allele frequency fell below 0.4 or above 0.6. This was performed to filter down to clonal-only mutations to facilitate comparisons with muver.

Overall run time for each tool was assessed with three separate data sets selected to represent a range of sequencing depths and mutation counts. The time to generate processed alignment files was added to the runtime for each program. VarScan’s total also includes the time required to produce the necessary input files in mpileup format, while MuTect2’s total includes the time required to perform base quality score recalibration. As multithreading is not supported by mpileup or VarScan itself, samples were processed concurrently, with only the longest run time for each stage of the analysis included in the total. Though the option for multithreaded operation exists for MuTect2 and the GATK components utilized for BQSR, attempts to run these tools with multiple central processing unit (CPU) threads did not complete successfully without errors. To resolve this issue, the MuTect2 analyses were also performed for all samples concurrently, utilizing a single CPU thread. Muver’s total includes time required for any necessary depth correction and subsequent re-calling of mutations.

### Estimating false negative indel rates in previous work

In previous work, indel rates were corrected for one known false negative effect: reads that add depth but not useful indel information. Depending on the length of a repeat tract, some fraction of reads will partially traverse that tract. These uninformative reads may drive indel variants below hard allelic fraction cutoffs, resulting in false negative or type II statistical errors [9]. If *L* is the repeat tract length, *D* is the average coverage depth, *R* is the read length, and *C* is the allelic fraction cutoff, then the false negative rate, *B*(*L*;*D*,*p*), should be

$$ B\left(L;D,p\right)=\sum \limits_{x=0}^L\left(\genfrac{}{}{0pt}{}{D\times C}{L}\right){p}^x{\left(1-p\right)}^{D\times C-x} $$

where *p* is the fraction of reads that cross the entire homopolymer,

$$ p=\left(R-2\left(L+\delta L\right)\right)/\left(R-\left(L+\delta L\right)\right). $$

Indel rates were corrected by dividing by 1-*B*(*L*;*D*,*p*), under the assumption that the false positive (type I error) rate was near zero, as evidenced by a lack of indels called in *wild type* samples.

### Calculating mutation rates

Briefly, as per [18], the mutation rate, per base pair per generation, for mutation type *i*, in bin *b*, is

$$ {\mu}_{bp,i,j}=\frac{N_{i,b,j}}{N_{bp,b}\times {gen}_{tot,j}}, $$

where *N*_*bp*, *b*_ is the number of base pairs in bin *b*. The number of mutations of type *i* in bin *b* (*N*_*i*, *b*, *j*_; accounting for ploidy) and the number of generations over which mutations accumulated (*gen*_*tot*, *j*_) are both summed across kindred isolates of strain *j* unless otherwise noted. *N*_*bp*, *b*_ is set to the global ploidy of strain *j* (usually 2) for the special case of whole genome rates (*μ*_*bp*, *i*, *j*_ = *μ*_*g*, *i*, *j*_).

### Simulation of multi-base deletions in a/T homopolymers

Monte Carlo simulations of the accumulation of multi-base deletions in A/T homopolymers were performed based on aggregate rates derived from *pol2*-M644G *msh2Δ* and *pol3*-L612 M *msh2Δ* samples. Rates were calculated assuming all multi-base insertions or deletions represented a series of single-base events. Simulations were run for 684 and 675 generations, the respective means for all *pol2*-M644G *msh2Δ* and *pol3*-L612 M *msh2Δ* outgrowth samples, and 5000 iterations were performed in each case. For each generation, the possibility of a single-base insertion, single-base deletion, and no change were considered within all A/T homopolymers of length 7-18 genome-wide.

## Results

The sensitivity and false positive rates (FPRs) of muver were determined by analyzing clonal differences between Illumina sequencing data for the father and son from the Genome in a Bottle Consortium (GiaB) Ashkenazim Trio. The same data was analyzed with GATK HaplotypeCaller v3.7 and VarScan v2.4.3 for comparison. Muver was then used to identify clonal mutations in previously collected *Saccharomyces cerevisiae* whole genome mutation accumulation experiments [18]. Strains with mutations in replicative DNA polymerases α, δ or ε (*pol1-L868 M*, *pol3-L612 M* or *pol2-M644G*, respectively), with or without homozygous deletion of essential mismatch repair (MMR) gene *MSH2*, were compared to wild-type strains. Muver compared each outgrowth sample to an ancestral sample (t0) collected at the beginning of the experiment. Mutation rates were calculated based on the number of elapsed generations. Muver’s results were compared to those of MuTect2, VarScan, rates from reporter gene experiments, and the analysis pipeline previously used to call clonal mutations (hereafter referred to as pipe2015). Muver results compared favorably to all other pipelines and revealed an unforeseen feature of replication infidelity in repeat tracts.

### Muver sensitivity and false positive rate with human data

Analysis of the GiaB father and son samples was performed with muver using family-wise error rates (FWER) from 2 × 10^− 7^ to 0.9. Sensitivity and FPRs were calculated considering only high-confidence GiaB calls. For the examined FWER range, the sensitivity and false positive rate increase approximately linearly for both substitutions and indels, with no clear inflection points to suggest an optimal FWER (Additional file 1: Figure S5). Based on these observations, as well as those in the analysis of yeast experiments during muver’s development, we selected 0.01 as the default FWER, electing to reduce the false positive rate at the expense of sensitivity.

**Fig. 2:**
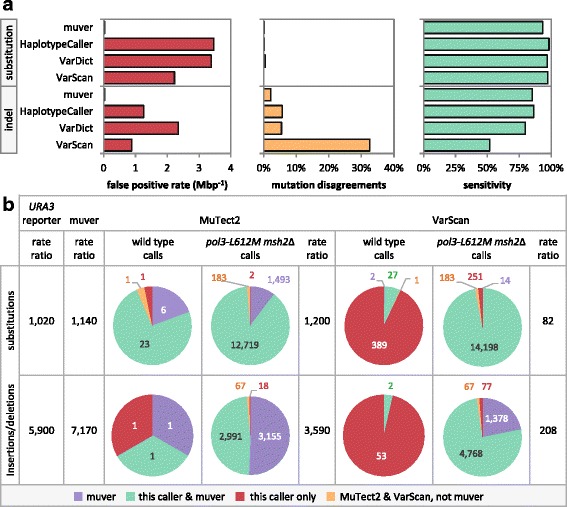
Comparison of muver results with other common mutation calling software. **a** Muver compared to GATK HaplotypeCaller, VarScan, and VarDict mutation calls in a Genome in a Bottle (GiaB) human father-son pair. Presumed false positives are mutations called where none are implied by GiaB genotype calls. Mutation disagreements are mutation calls with positions that agree but mutation identities that disagree with GiaB. Sensitivity is the percent of GiaB genotype differences identified by a given caller, excluding loci with identity disagreements. False positive rates (FPR) are per million base pairs (Mbp^− 1^) tested by both GiaB and a given caller (i.e. excluding areas of excessively low coverage, etc.). **b** Comparison of clonal substitution and insertion/deletion calls between muver, MuTect2 and VarScan for *Saccharomyces cerevisiae* with very low (*wild type*) and very high (*pol3-L612 M msh2*Δ) mutation rates. Mutations called by both MuTect2 and VarScan but not by muver are in orange. Mutation calls shared with muver are in green. Mutations called by MuTect2 or VarScan only are in red. Those called by muver but missed by the listed caller are in purple. Statistics from human data in panel A suggest that in panel B most red calls are false positives and most blue calls are false negatives. Mutation rate ratios (*pol3-L612 M msh2*Δ over *wild type*) for each caller were calculated from the number of called mutations, the size of the genome, and the number of elapsed cell divisions (see Methods). For comparison, mutation rate ratios from previous *URA3* assays are also presented. See Additional file 1: Table S1 for event counts

At the selected FWER, muver has FPRs of 0.036 and 0.030 Mbp^− 1^ for substitutions and indels, respectively (Fig. 2a, red), while still calling 93.6 and 87.3% of GiaB substitutions and indels, respectively. However, some muver calls (0.155 and 2.22%; Fig. 2a, orange) shared positions but not mutation types with GiaB calls, yielding more strict sensitivities of 93.4 and 85.1% (Fig. 2a, green), Most occurred where an allelic imbalance of more than threefold resulted in a best-fit genotype model with a possible subclonal mutation which was then rejected downstream due to insufficient statistical support.

For substitutions, muver displayed FPRs 96-, 94-, and 62-fold lower than those of GATK HaplotypeCaller [66], VarDict,[69] and VarScan [68], respectively (Fig. 2a, red), with only 5.0, 3.5, and 4.1% lower sensitivity (Fig. 2a, green). Similarly, for indels, muver displayed FPRs 42-, 79-, and 30-fold lower than those of HaplotypeCaller, VarDict, and VarScan (Fig. 2a, red), with far fewer GiaB mutation type disagreements (Fig. 2a, orange) and comparable to higher sensitivity (Fig. 2a, green).

Low false positive rates occurring with relatively modest losses in sensitivity are unique to muver. To achieve a substitution FPR comparable to that of muver run with default settings, HaplotypeCaller requires a calling confidence threshold of ~ 3000, and VarScan requires a somatic *p*-value threshold lower than 10^− 15^ (Additional file 1: Figure S5). Such conservative thresholds result in sensitivities of approximately 33 and 60%, respectively, compared to 94% for muver. For indels, thresholds > 2500 and < 10^− 10^ are necessary to match muver’s FPR, resulting in sensitivities below 26 and 66% for HaplotypeCaller and VarScan, while the rate for muver remains at a relatively high 87%. VarDict was unable to achieve false positive rates comparable to muver at any threshold, as it rounded any p-value lower than 10^− 5^ to zero. Sensitivity, however, varied little and remained relatively high regardless of the threshold applied, approximately 96 and 79% for substitutions and indels, respectively.

### Application to mutation accumulation studies

To assess muver’s performance in mutation accumulation experiments relative to other tools, we analyzed yeast *wild type* (*WT)* and *pol3-L612 M msh2*Δ samples with both VarScan [68] and MuTect2 [70] (Fig. 2b, Additional file 1: Table S1). Both VarScan and MuTect2 are designed to call mutations in matched tumor-normal pairs, however, their approaches to genotype and mutation calling differ from the methods employed by muver. VarScan relies on mpileup to provide observed allele counts, and no local reassembly of reads is performed. Mutations are called by comparing observed tumor and normal sample read counts using Fisher’s exact test, and genotypes are identified using defined allelic fraction thresholds. VarScan does not support identification of mutations for ploidies greater than two, and does not report more than a single alternate allele per position. MuTect2 utilizes the same local reassembly algorithm implemented in HaplotypeCaller, from which the read counts supporting each allele are derived. Mutations are identified based on a log odds score comparing the likelihood of observing the given read counts assuming a genuine mutation relative to the likelihood of the observation assuming no difference is present. Though MuTect2 supports arbitrary ploidy, only a single alternate allele is reported at each position, restricting the possible genotype calls to homozygous reference or heterozygous with a reported allelic fraction between 0 and 1.

For the *WT* mutation accumulation data set, muver, MuTect2, and VarScan called 31, 27, and 479 mutations, respectively. Under the conservative assumption that all mutations called were erroneous, these values provide upper bound FPR estimates in this system of 0.367, 0.320, and 5.68 Mbp^− 1^ for muver, MuTect2, and VarScan, respectively. For muver and VarScan these are higher than measured using GiaB data.

To assess FPRs in a similar context, but more directly, we called mutations after splitting reads from a single sample (TAK137) into virtual outgrowth and t0 samples, using the “virtual tumor” method as applied by the authors of MuTect [70]. As reads were derived from the same source, no mutations should be called. Of the three tools, only VarScan reported mutations passing all filters (a total of 9). The *WT* mutation accumulation and virtual outgrowth/control results both suggest low FPRs for muver and MuTect2. We performed a second virtual outgrowth/control analysis using the *pol3-L612 M msh2Δ* outgrowth TAK280, a sample expected to harbor a very large number of variant sites. Again, there were zero false positive calls in the muver and MuTect2 results, and 9 for VarScan. Assuming 900 generations of mutation accumulation, VarScan’s 9 false positives would imply a mutation rate per base per yeast cell division equal to or greater than the rate for any wild type eukaryote yet assessed through whole genome mutation accumulation (rates averaged if multiple experiments available for a given species [9, 13, 18–47]). This illustrates the importance of very low FPRs when calculating rates from mutation accumulation experiments. Muver has not been designed to detect rare variants in heterogeneous tumor samples, the domain of other tools designed for that purpose such as MuTect2. Muver, if used for such would detect only mutations in the last common ancestor of the bulk tumor. Muver would be more appropriate for mutation accumulation experiments in cancer cell lines or tumor organoids.

In contrast to the *WT* strain, the mutation rate for the *pol3-L612 M msh2*Δ background is elevated by a mutator variant of DNA Polymerase δ and by the absence of mismatch repair (MMR) [18, 24, 59, 71, 72]. A total of 21,239 mutations were called across all tools for the four *pol3-L612 M msh2*Δ outgrowth samples (Figure 2b, Additional file 1: Table S1). The three methods agreed on the positions of over 70% of mutation calls; 16% were identified by muver and VarScan, but not MuTect2. Most of these sites show evidence of non-reference alleles in the t0 sample or more than one alternate allele present in the outgrowth sample. MuTect2 explicitly excludes the former and applies “triallelic_site” or “homologous_mapping_event” flags to the latter. An additional 1% were called by MuTect2 and VarScan, but not muver, however, nearly all of these were excluded from consideration due to abnormally high or low depth in the t0 or outgrowth sample. Sites called by MuTect2 alone or by MuTect2 and muver together represent less than 1% of calls. The calls by VarScan alone were more frequent at ~ 3%. Approximately two-thirds of these are excluded by muver (insufficient t0 versus outgrowth difference; filtered due to insufficient/abnormal depth or t0 subclonal strand bias, thus likely due to mismapping) and for most of the remainder, muver calls identical clonal genotypes for the outgrowth and t0 samples after accounting for subclonal populations or mismapping issues in one or both samples.

Nearly all muver-specific mutations, the remaining 6% of calls, were indels within repeat tracts. These are mutation types and contexts that muver was specifically designed to target. Given much lower mutation rates in *WT* samples than in *pol3-L612 M msh2*Δ but presumably the same rates of PCR error and mismapping, these calls appear to be genuine.

Observed run times for each assessed tool varied widely depending on sample number and read depth, with VarScan’s analyses completing in 3.5 to 10 h and MuTect2’s completing in 1.5 to 7.5 days. Muver fell between these two extremes, requiring 5.5 to 15 h (Additional file 1: Table S2), representing a > 6-fold reduction relative to MuTect2, but a 1.5-fold increase relative to VarScan. These analyses were performed using up to 20 CPUs when parallel processing was possible, however, the total CPU time required follows a similar trend, with muver performing somewhat less efficiently than VarScan, but more efficiently than MuTect2.

### Muver compared to previous single locus results

**Fig. 3:**
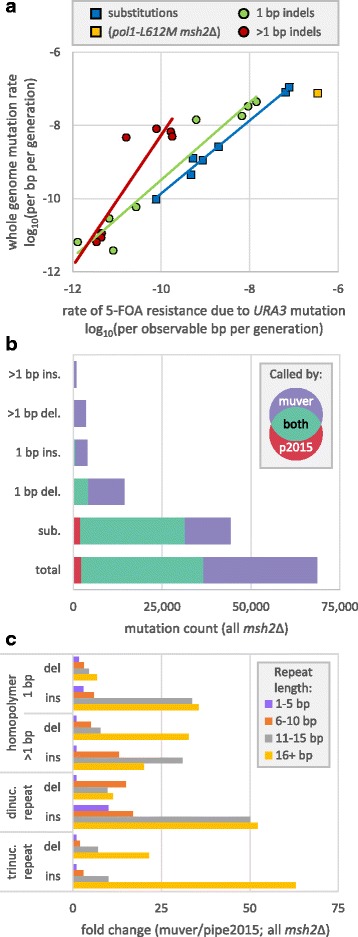
Comparison of muver calls with previous *Saccharomyces cerevisiae* mutation accumulation experiments. **a** Log-transformed substitution (square) and indel (circle) mutation rates, per base pair per generation, determined across the genome by muver of by acquisition of resistance to 5-fluoroorotic acid (5-FOA) upon mutation of the *URA3* reporter gene [63]. Solid lines are linear regressions for the log-transformed rates (*R*^*2*^ = 0.988 and 0.908 for substitutions and single-base indels, blue and green, respectively; excluding pol1-L868 M msh2Δ substitutions in yellow; *R*^*2*^ = 0.758 for multi-base indel rates in red). **b** Mutation counts from muver (purple) and pipe2015 (red; [18]) for *MSH2*-deficient *S. cerevisiae* presented here. Counts for mutations called by both pipelines are shown in green. **c** Fold-change in mutation counts (muver over pipe2015) in repeat tracts of 1-5 bp (purple), 6-10 bp (orange), 11-15 bp (grey) and more than 15 bp (gold). In calculating fold changes, pseudocounts were added to the numerator and denominator for categories where the pipe2015 totals were zero. Abbreviations: del. = deletions; ins. = insertions; dinuc. = dinucleotide repeats; trinuc. = trinucleotide or triplet repeats

Until the advent of whole genome mutation accumulation, mutation reporter assays, specifically forward mutation assays, were the gold standard for measuring mutation rates and were often extrapolated to estimate overall rates across the genome. Whole genome substitution and single-base indel rates called by muver yield ratios of *pol3-L612 M msh2*Δ and *WT* mutations consistent with those previously reported from the *URA3* reporter system [62, 63] (Fig. 2b), after accounting for higher indel rates outside of genes in the absence of MMR [9, 18]. Ratios from muver match the reporter data better than those of MuTect2 and VarScan. When comparing mutation rates, the former illustrates the need for sensitivity (see indels in Fig. 2b) and the latter illustrates the need for low FPRs. Overall there is good correlation between muver whole genome calls and mutation rates from the reporter assay (Fig. 3a). The correlations between log-transformed rates are linear over at least a three-order-of-magnitude dynamic range (per base pair per generation; Fig. 3a; *R*^*2*^ = 0.988 and 0.933 for substitutions and single-base indels, respectively; excluding *pol1-L868 M msh2*Δ substitutions). Mutation accumulation substitution and single-base indel rates are respectively 1.43 and 6.08-fold higher on average (excluding *pol1-L868 M msh2*Δ substitutions). The latter was expected given higher predicted indel rates in long homopolymers of the sorts not found in *URA3* [9]. The mutation accumulation substitution rate in *pol1-L868 M msh2*Δ samples reported by muver is about 5-fold lower than in the reporter system. Thus, *URA3* is not a representative model of unrepaired base-base mismatches made by Pol α-L868 M.

Muver-called and *URA3* reporter multi-base indel rates are less correlated (*R*^*2*^ = 0.813; Fig. 3a). On average, the reporter assay underestimates the whole genome multi-base indel rate by 2.2 and 118-fold in MMR-proficient and MMR-deficient strains, respectively. *URA3* appears to be a poor model for MMR-repairable multi-base indel loops. Again, this was expected given higher predicted indel rates in long repeat tracts, that are common in the genome but rare in *URA3* [9]. This suggests that whole genome mutation accumulation experiments are more suitable for studying processes for which multi-base indels are diagnostic (i.e. microsatellite instability in MMR-deficient tumors [73, 74]).

Correlations between muver calls and *URA3* mutations rates inform muver FPR estimates. Pipe2015 identified only eight indels in the 28 MMR-proficient samples, all single-base and all from strains with either Pol δ or ε mutator variants [18]. Muver identified two indels in the seven wild type samples and two in the six Pol α-variant samples. If these thirteen samples are considered negative controls, then given the 12 Mbp *cerevisiae* genome, the upper bound for the indel FPR is ≤0.026 Mbp^− 1^, remarkably similar to the value measured in the comparison of human genomes. From a regression of mutation accumulation versus *URA3* single-base indel rates (Fig. 3a), the expected count for these thirteen samples is 3.5. The observed count is not significantly greater than this value (Poisson *p* = 0.28), suggesting that the apparent upper bound indel FPR is overestimated. Muver found 28 single-base (up from eight) and five multi-base indels (up from zero) in the fifteen MMR-proficient Pol δ and ε mutator samples, a seven-fold increase over the maximum muver indel FPR.

### Muver compared to previous whole genome mutation accumulation results

A major deficiency of previous analyses was that reads that overlap but do not fully traverse a given repeat tract provide no information on changes in tract length but were nonetheless included in local coverage calculations. Longer tracts and shorter reads mean a higher likelihood that the apparent allelic fraction of an indel will fall below filter thresholds. However, simply lowering filter thresholds would also increase false positive rates. Our previous solution was to accept the high false negative rate for pipe2015 and then adjust the indel rate calculation based on a statistical model of this error source [18]. This method yielded high certainty for indel rates in common (i.e. short) repeat tracts, but the rationale was more tenuous for very rare tracts where extrapolation could magnify stochastic variation.

Muver identifies more mutations of all classes than did pipe2015 [18]. Excluding those now believed to be pipe2015 false positives, overall clonal mutation counts among 19 outgrowths lacking *MSH2* increased by 90%, to over 66,000, including a 40% increase in base pair substitutions (Fig. 3b). Muver increases indel calls 4.3-fold, however, the reported calling rate increases more for some indels than for others. Increases are greater for insertions and multi-base indels than for deletions and single-base indels. In outgrowths lacking *MSH2*, single-base deletion counts increase 3.3-fold (4274 to 14,143) while multi-base insertion counts increase 42-fold (20 to 834).

Muver increases counts more substantially in longer repeat tracts compared to shorter, as would be expected from previous statistical projections [9], and in general, insertion counts more than deletion counts (Fig. 3c). The greatest fold changes were observed for the longest insertions and deletions within the longest repeat tracts (Additional file 1: Figure S6). The net result is that muver, on one extreme, increases single-base deletion counts in short 1-5 bp homopolymers 1.7-fold, and on the other, increases trinucleotide repeat insertion counts 63-fold for tracts longer than 16 bp.

**Fig. 4:**
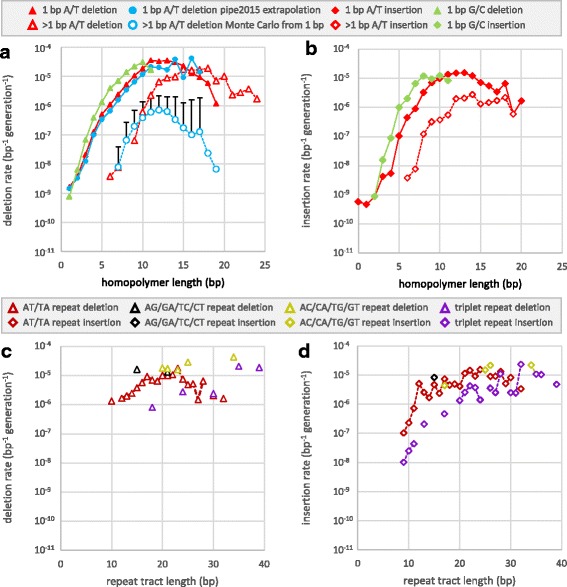
Muver-derived insertion and deletion rates in repeat tracts in pol3-L612 M msh2Δ samples. Rates (per bp per generation) of single-base (filled) and multi-base (open) deletions (triangles) and insertions (diamonds) are shown versus tract length for repeats with unit length of 1 (homopolymers; **a-b**) or greater than 1 (dinucleotide and triplet repeats; **c-d**). See Additional file 1: Figure S7 for rates in *pol2-M644G msh2*Δ samples

For contexts in which previous extrapolations were presented with high confidence [9], such as A/T homopolymers of less than 11 bp, muver-derived indel rates correspond well to those projections (e.g. Fig. 4a-b and Additional file 1: Figure S7A-B). This implies that muver has corrected the calling deficiencies accounted for in those extrapolations. No robust multi-base indel curves (versus tract length) were constructible from pipe2015 calls for any yeast genotype besides multi-base deletions in A/T homopolymers, and these were considered unreliable. In contrast, muver found enough to construct multi-base A/T insertion, dinucleotide deletion and insertion, and trinucleotide deletion and insertion curves for Pol δ and ε mutator variants lacking *MSH2* (e.g. Fig. 4c-d and Additional file 1: Figure S7C-D).

### Insights into DNA replication fidelity

Previous statistical extrapolations of indel rates inflated uncertainty in homopolymers of greater than 13 bp. Though it appeared that multi-base deletion rates in long A/T homopolymers exceeded what would be expected for sequential single-base deletions, the difference was insignificant [9]. However, using muver-derived rates, Monte Carlo simulations suggest that single-base A/T deletion rates are sufficient to explain multi-base A/T deletion rates in runs of up to 10 bp, but thereafter, multi-base deletion rates exceed the 95% confidence interval from the simulations, initially by a few-fold but eventually (in > 15 bp tracts) by two orders of magnitude or more. In fact, multi-base rates exceed single-base rates in tracts of > 15 bp.

Statistical extrapolations suggested that indel rates in long runs are largely independent of the polymerase variants present, attributed to a lack of proofreading once repeat tracts extend past the polymerase footprint [9, 13]. This conclusion was based on single-base A/T deletion and insertion rates which were projected to plateau at roughly 10^− 5^ and 10^− 6^ per base pair per generation, respectively. Though there is noise, muver-derived rates plateau between 10^− 5^ and 10^− 6^ per base pair per generation for single-base insertions and multi-base indels of all kinds in all long repeat contexts, regardless of polymerase status or repeat context.

## Discussion

The muver framework combines common variant discovery workflows with novel methods (Fig. 1) to improve identification of mutations in difficult genomic contexts and has been optimized for accurate and sensitive mutation calling in lineal samples. Muver employs GATK HaplotypeCaller to identify potential reference variants and to calculate strand-specific read depths. Muver models genome-wide read depths and excludes regions consistent with poor read mapping. Muver further identifies and excludes regions that significantly exceed the average global read depth, minimizing false positive calls. Muver can accommodate arbitrary ancestral and descendant ploidies and supports user-definition of local copy number variants (CNVs). Muver identifies mutations by comparing read counts per strand and per allele between ancestral and descendant samples. Sites that differ between ancestor and descendant are compared to genotype models that account for observed alleles, assumed ploidy, empirical error rates, and the potential for subclonal mutations across a range of population fractions. The latter also helps correct for allelic imbalances introduced through Bayesian haplotype reassignment performed by HaplotypeCaller and through library contamination (see Additional file 2).

In the current implementation of the muver framework, contamination and error are controlled incidentally, detected in the course of identifying subclonal mutations, however, minor modification would support explicit control. For example, in the application of the muver framework to matched tumor/normal comparisons, a user-specified normal tissue contamination fraction could be incorporated into the expected rates of all potential genotypes when performing tumor sample genotype calling. The flexibility of the genotype and mutation calling methods implemented within the muver framework will allow many systematic sources of error in allelic fractions to be modeled and controlled.

Muver identifies mutation events by considering all possible mutations that may occur given observed alleles. Given called t0 control and outgrowth genotypes, muver then enumerates all possible sequences of mutations and determines mutations that must necessarily have occurred, and notes any ambiguities. As triploid and tetraploid cells are common in yeast, muver also makes considerations for ploidy. Unlike other approaches, muver explicitly considers ploidy during genotype and mutation calling, allowing direct comparison of samples with different ploidy. By default, muver excludes regions with depths that differ significantly from the global average, but substitutions and indels are considered simultaneously with CNVs when local copy numbers are provided. Failure to account for ploidy/copy number or to filter regions of unknown copy number can result in an increased false positive rate, or the misinterpretation of true mutations as noise. Accurate understanding of these features is crucial for the interpretation of data derived from experiments where outgrowth samples can acquire many copy number variations and even global changes in ploidy. Ploidy changes are common in cancers [75], correlate with the total number of mutations and affect cell proliferation and immune evasion [76].

Muver performs favorably when compared to other commonly used tools. When analyzing human Genome in a Bottle data (Fig. 2a), muver exhibited far fewer mutation type disagreements than did VarScan, VarDict, and GATK HaplotypeCaller, 62- to 96-fold lower substitution FPRs, and 30- to 79-fold lower indel FPRs. When analyzing both GiaB human data and previous yeast mutation accumulation data (Fig. 2a), muver has higher indel sensitivity and a lower indel FPR than VarScan. In the same yeast data, muver had higher indel and substitution sensitivity than MuTect2 with comparable FPRs. These performance improvements are the result of detailed empirical analysis of read coverage performed by muver, as well as the per-sample assessment of sequencing error that informs the models utilized for per-locus genotype calling. These models incorporate ploidy and homogeneity assumptions that follow from accumulation experiments or other ancestor/progeny comparisons, while VarScan utilizes simple thresholds for genotype calling, and MuTect2 is designed for the detection of low frequency mutations, requiring aggressive filtering to ensure only clonal changes are reported. Overall run times for muver were substantially lower than for MuTect2, while not dramatically higher than VarScan. Given the relative sensitivity and specificity of each tool, muver represents the best balance of accuracy and computational performance and is a practical option for regular use in whole-genome mutation accumulation analyses.

Muver results compare well with previous studies and reveal new information. Muver-derived indel calling rates more closely resemble predicted indel mutation rates than calls made previously [9]. Substitution rates derived with muver correlate well with previous single locus reporter assays, while single-base indel calls conform well with previous high confidence statistical extrapolations. Muver-identified multi-base indels exceed those from previous yeast mutation accumulation assays, revealing unprecedented details for indels in long repeat tracts. Multi-base indels occur in mismatch repair-deficient yeast in long homopolymers at rates that exceed what would be expected from sequential single-base indels (open circles versus open triangles in Fig. 4a and Additional file 1: Figure S7A). Multi-base A/T deletion rates eventually exceed the rates of single A/T deletions (open red triangles versus closed red triangles in Fig. 4a and Additional file 1: Figure S7A). Multi-base indel rates in homopolymers closely match multi-base indel rates in di- and trinucleotide repeats. These effects are independent of mutator polymerase background but begin when homopolymer length exceeds the polymerase footprint. Taken together, this suggests an unforeseen model wherein polymerases slip multiple bases in one homopolymer synthesis reaction, as if the repeat unit in long homopolymers is greater than one base pair. Thus, multi-base homopolymer indels should not be considered multiple single-base indels when analyzing such effects as microsatellite instability.

## Conclusions

The muver framework introduces novel methods in genotype calling, identification of mutations, and inference of mutation identity. Detailed, per-sample, observation of sequencing data allows unsupervised filtering and error correction, without the need for precise tuning of thresholds. Muver is capable of accurate and sensitive calling of clonal mutations, particularly of heretofore difficult indels, and supports analysis of complex genomic contexts involving ploidy differences unavailable in other packages. Muver’s demonstrated utility in the identification of indels and substitutions, from yeast to humans, makes it applicable to a variety of studies that could illuminate the evolutionary, mechanistic and medical implications of mutagenesis.

## Additional files

Additional file 1: Supplemental Text and Figures. Contains 8 supplemental tables and 7 supplemental figures. (DOCX 2454 kb)

Additional file 2: Results Files. Zip file containing VCF and TXT formatted muver results for all yeast data sets analyzed. (ZIP 10697 kb)

## Abbreviations

5-FOA: 5-fluoroorotic acid
BQSR: Base quality score recalibration
CNV: Copy number variation
CPU: Central processing unit
FPR: False positive rate
FWER: Family-wise error rate
GATK: Genome analysis tool kit
GiaB: Genome in a bottle
HGVS: Human Genome Variation Society
indels: DNA insertions and deletions
MMR: DNA mismatch repair
muver: Our tool; stands for mutationes verificatae
NCBI: National Center for Biotechnology Information
PAC: Potential allelic conversion event
PCR: Polymerase chain reaction
pipe2015: Previous mutation calling pipeline
Pol α: DNA Polymerases α
Pol δ: DNA Polymerases δ
Pol ϵ: DNA Polymerases ϵ
RER: Ribonucleotide excision repair
t0: Initial time point, i.e. time point 0
WT: Wild type yeast strain
