# Supplementary material for: Muver, a computational framework for accurately calling accumulated mutations
Source: BMC Genomics. 2018 May 9;19:345. doi: 10.1186/s12864-018-4753-3 (PMC5944071; doi:10.1186/s12864-018-4753-3)
Supplement: Supplementary file 1 — Supplemental Text and Figures. Contains 8 supplemental tables and 7 supplemental figures. (DOCX 2454 kb) [file 12864_2018_4753_MOESM1_ESM.docx]

**SUPPLEMENTAL TABLES AND FIGURES**

|  | ***WT*** | | ***pol3*-L612M *msh2∆*** | |
| --- | --- | --- | --- | --- |
| *Called By* | *Common Sites* | *Matching Mutation Identity* | *Common Sites* | *Matching Mutation Identity* |
| muver Only | 2 | -- | 1,324 | -- |
| MuTect2 Only | 2 | -- | 20 | -- |
| VarScan Only | 479 | -- | 611 | -- |
| muver + MuTect2 | 0 | -- | 63 | 47 |
| muver + VarScan | 5 | 5 | 3,324 | 2,907 |
| MuTect2 + VarScan | 1 | 1 | 250 | 247 |
| All | 24 | 24 | 15,647 | 15,630 |

**Supplemental Table 1.**

Count of mutations called by all combinations of assessed tools. “Common Sites” are those where one or more tools indicates a mutation occurred at a given locus. “Matching Mutation Identity” indicates identical t0 and outgrowth genotypes were called by each tool.

|  |  |  | **Total Elapsed Time (min)** | | | **Total CPU Time (min)** | | |
| --- | --- | --- | --- | --- | --- | --- | --- | --- |
| *Data Set* | *Mean Depth per Sample* | *Called Mutations (muver)* | *VarScan* | *muver* | *MuTect2* | *VarScan* | *muver* | *MuTect2* |
| t0TAK147 *msh2∆* | 101 | 3,564 | 215 | 338 | 2,235 | 3,058 | 3,883 | 7,233 |
| t0TAK142 *pol3*-L612M | 341 | 197 | 612 | 891 | 8,248 | 8,005 | 8,102 | 27,234 |
| t0TAK163 *pol3*-L612M *msh2∆* | 287 | 21,059 | 467 | 912 | 11,155 | 7,589 | 12,697 | 37,851 |

**Supplemental Table 2.**

Total run time in minutes for assessed tools. Values vary by data set depending on sequencing depth and frequency of mutations. Prior to assessing muver’s performance, indices and repeat tract locations were generated once for the utilized reference FASTA file, with an elapsed time of 138 minutes.

| **Outgrowth Sample ID** | **Strain** | **Generation** | **t0 Sample** | **Substitutions** | **Indels** | **Total** |  |
| --- | --- | --- | --- | --- | --- | --- | --- |
| TAK258 | *msh2Δ* | 900 | TAK147 | 100 | 819 | 919 |  |
| TAK259 | *msh2Δ* | 900 | TAK147 | 127 | 1086 | 1213 |  |
| TAK260 | *msh2Δ* | 900 | TAK147 | 145 | 967 | 1112 |  |
| TAK262 | *msh2Δ* | 900 | TAK148 | 121 | 856 | 977 |  |
| TAK266 | *msh2Δ* | 660 | TAK147 | 52 | 268 | 320 |  |
| TAK118 | *pol1*-L868M | 900 | TAK138 | 20 | 1 | 21 |  |
| TAK119 | *pol1*-L868M | 900 | TAK138 | 16 | 1 | 17 |  |
| TAK121 | *pol1*-L868M | 900 | TAK139 | 15 | 1 | 16 |  |
| TAK123 | *pol1*-L868M | 900 | TAK139 | 21 | 1 | 22 |  |
| TAK72 | *pol1*-L868M | 900 | TAK138 | 18 | 0 | 18 |  |
| TAK73 | *pol1*-L868M | 900 | TAK139 | 28 | 0 | 28 |  |
| TAK165 | *pol1*-L868M *msh2Δ* | 900 | TAK149 | 5578 | 1005 | 6583 |  |
| TAK175 | *pol1*-L868M *msh2Δ* | 900 | TAK150 | 946 | 877 | 1823 |  |
| TAK216 | *pol1*-L868M *msh2Δ* | 900 | TAK149 | 3840 | 934 | 4774 |  |
| TAK264 | *pol1*-L868M *msh2Δ* | 900 | TAK149 | 5553 | 1060 | 6613 |  |
| TAK271 | *pol1*-L868M *msh2Δ* | 660 | TAK150 | 1836 | 805 | 2641 |  |
| TAK272 | *pol1*-L868M *msh2Δ* | 660 | TAK150 | 1624 | 802 | 2426 |  |
| TAK273 | *pol1*-L868M *msh2Δ* | 660 | TAK150 | 1222 | 843 | 2065 |  |
| TAK124 | *pol2*-M644G | 900 | TAK140 | 51 | 2 | 53 |  |
| TAK125 | *pol2*-M644G | 900 | TAK140 | 38 | 1 | 39 |  |
| TAK126 | *pol2*-M644G | 900 | TAK140 | 78 | 4 | 82 |  |
| TAK127 | *pol2*-M644G | 900 | TAK141 | 52 | 0 | 52 |  |
| TAK128 | *pol2*-M644G | 900 | TAK141 | 66 | 3 | 69 |  |
| TAK129 | *pol2*-M644G | 900 | TAK141 | 51 | 1 | 52 |  |
| TAK74 | *pol2*-M644G | 900 | TAK140 | 44 | 2 | 46 |  |
| TAK75 | *pol2*-M644G | 900 | TAK141 | 64 | 0 | 64 |  |
| TAK158 | *pol2*-M644G *msh2Δ* | 480 | TAK152 | 4450 | 1756 | 6206 |  |
| TAK166 | *pol2*-M644G *msh2Δ* | 660 | TAK151 | 2664 | 1781 | 4445 |  |
| TAK170 | *pol2*-M644G *msh2Δ* | 900 | TAK151 | 3923 | 1575 | 5498 |  |
| TAK180 | *pol2*-M644G *msh2Δ* | 480 | TAK152 | 3020 | 1845 | 4865 |  |
| TAK217 | *pol2*-M644G *msh2Δ* | 900 | TAK151 | 3708 | 1841 | 5549 |  |
| TAK130 | *pol3*-L612M | 900 | TAK142 | 41 | 3 | 44 |  |
| TAK131 | *pol3*-L612M | 900 | TAK142 | 44 | 7 | 51 |  |
| TAK132 | *pol3*-L612M | 900 | TAK142 | 49 | 2 | 51 |  |
| TAK134 | *pol3*-L612M | 900 | TAK143 | 55 | 2 | 57 |  |
| TAK135 | *pol3*-L612M | 900 | TAK143 | 54 | 1 | 55 |  |
| TAK76 | *pol3*-L612M | 900 | TAK142 | 48 | 3 | 51 |  |
| TAK77 | *pol3*-L612M | 900 | TAK143 | 54 | 2 | 56 |  |
| TAK215 | *pol3*-L612M *msh2Δ* | 900 | TAK163 | 4786 | 2206 | 6992 |  |
| TAK279 | *pol3*-L612M *msh2Δ* | 660 | TAK163 | 2564 | 1295 | 3859 |  |
| TAK280 | *pol3*-L612M *msh2Δ* | 660 | TAK163 | 3762 | 1823 | 5585 |  |
| TAK281 | *pol3*-L612M *msh2Δ* | 480 | TAK163 | 3196 | 1427 | 4623 |  |
| TAK112 | *WT* | 900 | TAK136 | 6 | 0 | 6 |  |
| TAK113 | *WT* | 900 | TAK136 | 6 | 2 | 8 |  |
| TAK115 | *WT* | 900 | TAK137 | 4 | 0 | 4 |  |
| TAK116 | *WT* | 900 | TAK137 | 3 | 0 | 3 |  |
| TAK117 | *WT* | 900 | TAK137 | 3 | 0 | 3 |  |
| TAK70 | *WT* | 900 | TAK136 | 5 | 0 | 5 |  |
| TAK71 | *WT* | 900 | TAK137 | 2 | 0 | 2 |  |

**Supplemental Table 3.**

List of examined outgrowth samples. Table includes paired t0 sample, count of observed substitutions and indels.

| Percent *WT* | Percent *pol3*-L612M *msh2∆* | Called Mutations | Proportion Relative to 100% *pol3*-L612M *msh2*∆ |
| --- | --- | --- | --- |
| 100 | 0 | 0 | 0 |
| 90 | 10 | 0 | 0 |
| 80 | 20 | 19 | 0.00315772 |
| 70 | 30 | 80 | 0.013295662 |
| 60 | 40 | 205 | 0.034070135 |
| 50 | 50 | 505 | 0.083928868 |
| 0 | 100 | 6017 | 1 |

**Supplemental Table 4.**

Assessment of miscalled mutations in samples with variable contamination. Reads derived from a *pol3*-L612M *msh2∆* sample were combined in listed proportions with a *WT* sample. Mutations were called using muver against an independent set of reads derived from the same *WT* sample.

| Family-wise Error Rate | Substitution Differences | muver only Substitutions | GiaB only Substitutions | Substitution FPR | Substitution Sensitivity | indel Differences | muver only indels | GiaB only indels | indel FPR | indel Sensitivity |
| --- | --- | --- | --- | --- | --- | --- | --- | --- | --- | --- |
| 9.00E-01 | 2,972 | 108 | 72,477 | 0.0465 | 0.9585 | 6,264 | 83 | 21,099 | 0.0357 | 0.8950 |
| 8.00E-01 | 2,960 | 105 | 74,259 | 0.0452 | 0.9575 | 6,234 | 83 | 21,663 | 0.0357 | 0.8929 |
| 7.00E-01 | 2,939 | 104 | 75,888 | 0.0448 | 0.9566 | 6,208 | 83 | 22,131 | 0.0357 | 0.8913 |
| 6.00E-01 | 2,942 | 103 | 77,364 | 0.0443 | 0.9558 | 6,183 | 82 | 22,675 | 0.0353 | 0.8893 |
| 5.00E-01 | 2,931 | 102 | 79,063 | 0.0439 | 0.9549 | 6,155 | 79 | 23,230 | 0.0340 | 0.8872 |
| 4.00E-01 | 2,922 | 100 | 80,973 | 0.0430 | 0.9539 | 6,120 | 78 | 23,863 | 0.0336 | 0.8849 |
| 3.00E-01 | 2,903 | 99 | 83,440 | 0.0426 | 0.9525 | 6,089 | 78 | 24,560 | 0.0336 | 0.8824 |
| 2.00E-01 | 2,868 | 95 | 86,895 | 0.0409 | 0.9506 | 6,047 | 77 | 25,561 | 0.0331 | 0.8787 |
| 1.00E-01 | 2,981 | 95 | 92,262 | 0.0409 | 0.9476 | 5,987 | 75 | 27,101 | 0.0323 | 0.8730 |
| 5.00E-02 | 2,929 | 93 | 98,907 | 0.0400 | 0.9440 | 5,935 | 71 | 28,802 | 0.0306 | 0.8667 |
| 1.00E-02 | 2,822 | 84 | 116,918 | 0.0362 | 0.9341 | 5,773 | 69 | 33,136 | 0.0297 | 0.8507 |
| 5.00E-03 | 2,762 | 83 | 126,194 | 0.0357 | 0.9291 | 5,691 | 67 | 35,130 | 0.0288 | 0.8434 |
| 1.00E-03 | 2,620 | 74 | 150,709 | 0.0318 | 0.9157 | 5,494 | 66 | 40,181 | 0.0284 | 0.8247 |
| 5.00E-04 | 2,549 | 74 | 162,820 | 0.0318 | 0.9091 | 5,407 | 63 | 42,494 | 0.0271 | 0.8162 |
| 1.00E-04 | 2,389 | 70 | 194,747 | 0.0301 | 0.8916 | 5,211 | 62 | 48,127 | 0.0267 | 0.7953 |
| 5.00E-05 | 2,313 | 68 | 210,112 | 0.0293 | 0.8832 | 5,129 | 62 | 50,680 | 0.0267 | 0.7858 |
| 1.00E-05 | 2,163 | 67 | 249,031 | 0.0288 | 0.8619 | 4,938 | 61 | 56,943 | 0.0263 | 0.7625 |
| 1.00E-06 | 1,840 | 61 | 311,076 | 0.0263 | 0.8279 | 4,673 | 61 | 66,517 | 0.0263 | 0.7268 |
| 2.00E-07 | 1,747 | 59 | 343,422 | 0.0254 | 0.8102 | 4,564 | 60 | 71,165 | 0.0258 | 0.7094 |

**Supplemental Table 5.**

Muver false positive rate (FPR) and sensitivity determined for a range of family-wise error rates. “Differences” refer to the count of loci where muver calls a mutation and the Genome in a Bottle (GiaB) high confidence genotype calls agree, but the genotypes called by muver were incorrect.

| Somatic p-value | Substitution Differences | VarScan only Substitutions | GiaB only Substitutions | Substitution FPR | Substitution Sensitivity | indel Differences | VarScan only indels | GiaB only indels | indel FPR | indel Sensitivity |
| --- | --- | --- | --- | --- | --- | --- | --- | --- | --- | --- |
| 1.00E-01 | 1,094 | 6,858 | 45,289 | 2.9384 | 0.9749 | 85,712 | 2,533 | 41,059 | 1.0853 | 0.5182 |
| 9.00E-02 | 1,092 | 6,296 | 45,300 | 2.6976 | 0.9749 | 85,669 | 2,379 | 41,102 | 1.0193 | 0.5182 |
| 8.00E-02 | 1,092 | 5,742 | 45,313 | 2.4602 | 0.9749 | 85,614 | 2,221 | 41,158 | 0.9516 | 0.5182 |
| 7.00E-02 | 1,092 | 5,201 | 45,322 | 2.2284 | 0.9749 | 85,546 | 2,070 | 41,226 | 0.8869 | 0.5182 |
| 6.00E-02 | 1,092 | 4,663 | 45,341 | 1.9979 | 0.9749 | 85,471 | 1,929 | 41,301 | 0.8265 | 0.5182 |
| 5.00E-02 | 1,092 | 4,145 | 45,363 | 1.7760 | 0.9748 | 85,392 | 1,783 | 41,380 | 0.7640 | 0.5182 |
| 1.00E-02 | 1,087 | 3,251 | 46,076 | 1.3929 | 0.9745 | 84,707 | 1,122 | 42,100 | 0.4807 | 0.5181 |
| 5.00E-03 | 1,083 | 2,863 | 46,500 | 1.2267 | 0.9742 | 84,471 | 1,017 | 42,372 | 0.4357 | 0.5180 |
| 1.00E-03 | 1,067 | 1,992 | 48,001 | 0.8535 | 0.9734 | 83,756 | 824 | 43,297 | 0.3531 | 0.5172 |
| 5.00E-04 | 1,056 | 1,789 | 48,742 | 0.7665 | 0.9730 | 83,389 | 757 | 43,823 | 0.3243 | 0.5166 |
| 1.00E-04 | 1,037 | 1,530 | 50,970 | 0.6556 | 0.9718 | 82,589 | 665 | 45,196 | 0.2849 | 0.5144 |
| 5.00E-05 | 1,028 | 1,444 | 52,094 | 0.6187 | 0.9712 | 82,223 | 629 | 45,918 | 0.2695 | 0.5130 |
| 1.00E-05 | 1,008 | 1,291 | 55,297 | 0.5531 | 0.9695 | 81,142 | 566 | 48,207 | 0.2425 | 0.5085 |
| 1.00E-06 | 979 | 1,109 | 61,127 | 0.4752 | 0.9664 | 79,125 | 494 | 52,998 | 0.2117 | 0.4979 |
| 1.00E-07 | 914 | 928 | 68,541 | 0.3976 | 0.9624 | 76,626 | 407 | 59,746 | 0.1744 | 0.4818 |
| 1.00E-08 | 817 | 765 | 78,097 | 0.3278 | 0.9573 | 73,859 | 331 | 67,946 | 0.1418 | 0.4611 |
| 1.00E-09 | 726 | 592 | 91,166 | 0.2537 | 0.9502 | 70,581 | 279 | 77,905 | 0.1195 | 0.4357 |
| 1.00E-10 | 631 | 467 | 109,869 | 0.2001 | 0.9402 | 67,177 | 217 | 89,140 | 0.0930 | 0.4060 |
| 1.00E-15 | 316 | 126 | 411,273 | 0.0540 | 0.7771 | 44,334 | 50 | 159,983 | 0.0214 | 0.2236 |
| 1.00E-20 | 234 | 56 | 1,114,778 | 0.0240 | 0.3961 | 18,052 | 14 | 226,072 | 0.0060 | 0.0723 |
| 1.00E-25 | 164 | 27 | 1,663,088 | 0.0116 | 0.0992 | 3,549 | 2 | 256,668 | 0.0009 | 0.0111 |
| 1.00E-30 | 86 | 6 | 1,826,739 | 0.0026 | 0.0106 | 314 | 1 | 262,658 | 0.0004 | 0.0007 |
| 1.00E-35 | 31 | 0 | 1,845,111 | 0.0000 | 0.0006 | 9 | 1 | 263,129 | 0.0004 | 0.0000 |
| 1.00E-40 | 7 | 0 | 1,845,995 | 0.0000 | 0.0002 | 0 | 1 | 263,146 | 0.0004 | 0.0000 |
| 1.00E-45 | 1 | 0 | 1,846,026 | 0.0000 | 0.0002 | 0 | 1 | 263,146 | 0.0004 | 0.0000 |
| 1.00E-50 | 0 | 0 | 1,846,030 | 0.0000 | 0.0002 | 0 | 1 | 263,146 | 0.0004 | 0.0000 |

**Supplemental Table 6.**

VarScan false positive rate (FPR) and sensitivity determined for a range of somatic p-value thresholds. “Differences” refer to the count of loci where VarScan calls a mutation and the Genome in a Bottle (GiaB) high confidence genotype calls agree, but the genotypes called by VarScan were incorrect.

| Calling Confidence Threshold | Substitution Differences | HC only Substitutions | GiaB only Substitutions | Substitution FPR | Substitution Sensitivity | indel Differences | HC only indels | GiaB only indels | indel FPR | indel Sensitivity |
| --- | --- | --- | --- | --- | --- | --- | --- | --- | --- | --- |
| 10 | 4,808 | 9,804 | 24,472 | 4.2007 | 0.9841 | 15,227 | 3,277 | 18,995 | 1.4041 | 0.8700 |
| 20 | 4,541 | 8,870 | 25,378 | 3.8005 | 0.9838 | 15,098 | 3,093 | 19,725 | 1.3252 | 0.8677 |
| 30 | 4,291 | 8,084 | 26,341 | 3.4637 | 0.9834 | 14,946 | 2,939 | 20,462 | 1.2593 | 0.8654 |
| 40 | 4,156 | 7,377 | 27,234 | 3.1608 | 0.9830 | 14,725 | 2,815 | 21,257 | 1.2061 | 0.8633 |
| 50 | 3,872 | 6,770 | 28,159 | 2.9007 | 0.9827 | 14,476 | 2,718 | 22,132 | 1.1646 | 0.8609 |
| 60 | 3,597 | 6,221 | 29,110 | 2.6655 | 0.9823 | 14,155 | 2,609 | 23,109 | 1.1179 | 0.8584 |
| 70 | 3,429 | 5,748 | 29,969 | 2.4628 | 0.9819 | 13,829 | 2,535 | 24,119 | 1.0862 | 0.8558 |
| 80 | 3,250 | 5,314 | 30,795 | 2.2769 | 0.9816 | 13,474 | 2,472 | 25,202 | 1.0592 | 0.8530 |
| 90 | 3,132 | 4,916 | 31,581 | 2.1063 | 0.9812 | 13,110 | 2,411 | 26,318 | 1.0330 | 0.8502 |
| 100 | 2,962 | 4,619 | 32,430 | 1.9791 | 0.9808 | 12,668 | 2,362 | 27,541 | 1.0120 | 0.8472 |
| 150 | 2,265 | 3,492 | 36,485 | 1.4962 | 0.9790 | 10,231 | 2,161 | 34,637 | 0.9259 | 0.8295 |
| 200 | 1,763 | 2,758 | 40,415 | 1.1817 | 0.9772 | 8,004 | 1,983 | 42,003 | 0.8496 | 0.8100 |
| 250 | 1,372 | 2,232 | 44,271 | 0.9563 | 0.9753 | 6,597 | 1,796 | 48,063 | 0.7695 | 0.7923 |
| 300 | 1,109 | 1,846 | 48,090 | 0.7909 | 0.9734 | 5,705 | 1,645 | 53,089 | 0.7048 | 0.7766 |
| 350 | 870 | 1,564 | 52,202 | 0.6701 | 0.9713 | 5,079 | 1,469 | 57,588 | 0.6294 | 0.7619 |
| 400 | 702 | 1,342 | 56,428 | 0.5750 | 0.9691 | 4,620 | 1,247 | 61,721 | 0.5343 | 0.7479 |
| 450 | 575 | 1,190 | 60,924 | 0.5099 | 0.9667 | 4,302 | 1,042 | 65,535 | 0.4465 | 0.7346 |
| 500 | 500 | 1,081 | 65,539 | 0.4632 | 0.9642 | 4,035 | 857 | 69,128 | 0.3672 | 0.7220 |
| 600 | 389 | 942 | 76,238 | 0.4036 | 0.9585 | 3,591 | 580 | 75,721 | 0.2485 | 0.6986 |
| 700 | 318 | 810 | 89,696 | 0.3471 | 0.9512 | 3,206 | 446 | 81,750 | 0.1911 | 0.6772 |
| 800 | 291 | 707 | 107,690 | 0.3029 | 0.9415 | 2,846 | 396 | 87,698 | 0.1697 | 0.6559 |
| 900 | 274 | 641 | 131,967 | 0.2746 | 0.9284 | 2,589 | 353 | 93,691 | 0.1512 | 0.6341 |
| 1,000 | 256 | 575 | 165,342 | 0.2464 | 0.9103 | 2,321 | 326 | 100,038 | 0.1397 | 0.6110 |
| 1,500 | 215 | 326 | 515,974 | 0.1397 | 0.7204 | 1,411 | 199 | 134,506 | 0.0853 | 0.4835 |
| 2,000 | 176 | 196 | 969,022 | 0.0840 | 0.4751 | 864 | 136 | 170,175 | 0.0583 | 0.3500 |
| 2,500 | 142 | 131 | 1,185,614 | 0.0561 | 0.3578 | 570 | 83 | 195,474 | 0.0356 | 0.2550 |
| 3,000 | 100 | 89 | 1,235,354 | 0.0381 | 0.3309 | 375 | 55 | 208,302 | 0.0236 | 0.2070 |
| 3,500 | 65 | 61 | 1,257,374 | 0.0261 | 0.3190 | 250 | 40 | 214,666 | 0.0171 | 0.1833 |
| 4,000 | 38 | 50 | 1,290,477 | 0.0214 | 0.3010 | 163 | 32 | 219,601 | 0.0137 | 0.1649 |
| 4,500 | 19 | 40 | 1,343,634 | 0.0171 | 0.2723 | 114 | 26 | 224,268 | 0.0111 | 0.1473 |
| 5,000 | 10 | 36 | 1,417,096 | 0.0154 | 0.2325 | 84 | 25 | 229,278 | 0.0107 | 0.1284 |
| 6,000 | 6 | 28 | 1,592,797 | 0.0120 | 0.1373 | 44 | 21 | 240,066 | 0.0090 | 0.0875 |
| 7,000 | 3 | 25 | 1,743,942 | 0.0107 | 0.0555 | 23 | 14 | 249,806 | 0.0060 | 0.0506 |
| 8,000 | 2 | 21 | 1,820,890 | 0.0090 | 0.0138 | 11 | 14 | 256,961 | 0.0060 | 0.0235 |
| 9,000 | 2 | 20 | 1,842,191 | 0.0086 | 0.0022 | 5 | 13 | 261,184 | 0.0056 | 0.0074 |
| 10,000 | 2 | 20 | 1,845,537 | 0.0086 | 0.0004 | 1 | 11 | 262,767 | 0.0047 | 0.0014 |

**Supplemental Table 7.**

HaplotypeCaller (HC) false positive rate (FPR) and sensitivity determined for a range of calling confidence thresholds. “Differences” refer to the count of loci where HaplotypeCaller identifies different genotypes for the father and son sample, and the Genome in a Bottle (GiaB) high confidence genotype calls agree, but those called by HaplotypeCaller were incorrect.

| p-value Threshold | Substitution Differences | VarDict only Substitutions | GiaB only Substitutions | Substitution FPR | Substitution Sensitivity | indel Differences | VarDict only indels | GiaB only indels | indel FPR | indel Sensitivity |
| --- | --- | --- | --- | --- | --- | --- | --- | --- | --- | --- |
| 0.1 | 9,813 | 9,359 | 46,216 | 4.0100 | 0.9697 | 14,411 | 6,919 | 39,066 | 2.965 | 0.797 |
| 0.09 | 9,805 | 9,050 | 46,286 | 3.8776 | 0.9696 | 14,408 | 6,649 | 39,072 | 2.849 | 0.797 |
| 0.08 | 9,801 | 8,756 | 46,355 | 3.7516 | 0.9696 | 14,405 | 6,359 | 39,080 | 2.725 | 0.797 |
| 0.07 | 9,799 | 8,492 | 46,438 | 3.6385 | 0.9695 | 14,400 | 6,072 | 39,091 | 2.602 | 0.797 |
| 0.06 | 9,793 | 8,193 | 46,589 | 3.5104 | 0.9695 | 14,394 | 5,799 | 39,111 | 2.485 | 0.797 |
| 0.05 | 9,783 | 7,900 | 46,758 | 3.3849 | 0.9694 | 14,387 | 5,476 | 39,129 | 2.346 | 0.797 |
| 0.04 | 9,770 | 7,569 | 47,002 | 3.2431 | 0.9693 | 14,377 | 5,143 | 39,150 | 2.204 | 0.797 |
| 0.03 | 9,755 | 7,193 | 47,338 | 3.0819 | 0.9691 | 14,372 | 4,788 | 39,173 | 2.051 | 0.797 |
| 0.02 | 9,735 | 6,831 | 47,914 | 2.9268 | 0.9688 | 14,360 | 4,373 | 39,226 | 1.874 | 0.796 |
| 0.01 | 9,694 | 6,401 | 49,052 | 2.7426 | 0.9682 | 14,340 | 3,881 | 39,332 | 1.663 | 0.796 |
| 0.005 | 9,653 | 6,164 | 50,319 | 2.6411 | 0.9675 | 14,318 | 3,624 | 39,440 | 1.553 | 0.796 |
| 0.001 | 9,482 | 5,777 | 53,708 | 2.4752 | 0.9658 | 14,256 | 3,244 | 39,808 | 1.390 | 0.795 |
| 0.0005 | 9,422 | 5,632 | 55,279 | 2.4131 | 0.9650 | 14,225 | 3,115 | 39,994 | 1.335 | 0.794 |
| 0.0001 | 9,266 | 5,290 | 59,010 | 2.2666 | 0.9630 | 14,118 | 2,871 | 40,540 | 1.230 | 0.792 |
| 0.00005 | 9,114 | 5,118 | 60,770 | 2.1929 | 0.9621 | 14,034 | 2,745 | 40,884 | 1.176 | 0.791 |
| 0.00001 | 8,596 | 4,712 | 66,615 | 2.0189 | 0.9593 | 13,739 | 2,501 | 42,269 | 1.072 | 0.787 |
| 0.000001 | 8,596 | 4,712 | 66,615 | 2.0189 | 0.9593 | 13,739 | 2,501 | 42,269 | 1.072 | 0.787 |

**Supplemental Table 8.**

VarDict false positive rate (FPR) and sensitivity determined for a range of p-value thresholds. “Differences” refer to the count of loci where VarDict identifies different genotypes for the father and son sample, and the Genome in a Bottle (GiaB) high confidence genotype calls agree, but those called by VarDict were incorrect.


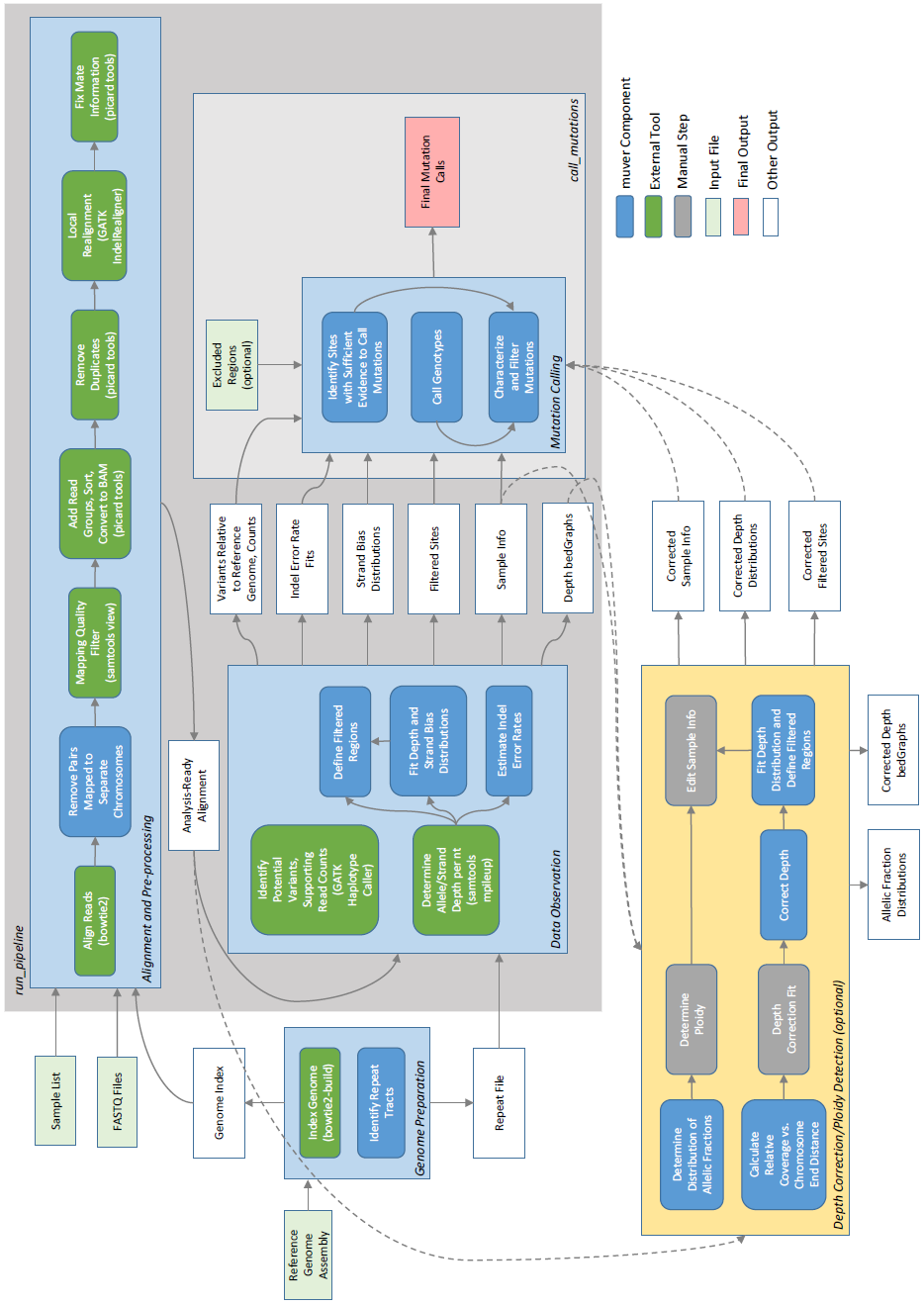


**Supplemental Figure 1.**

Detailed overview of muver operation. Input files: reference genome assembly (FASTA), sequenced reads (FASTQ), tab-delimited text file associating FASTQ files with sample identifiers; optionally, a list of excluded regions may be provided where genotypes and mutations will not be called. Output files: called mutations in VCF format and a tab-delimited text file including detailed information about each site examined. *Genome Preparation*: generate alignment index and identify repeat tracts genome-wide. *Alignment and Pre-processing*: align reads, filter, and perform indel realignment; performed primarily with standard, publicly available tools. *Data Observation*: identify sites with potential to vary from the reference, and extract counts of supporting reads with GATK HaplotypeCaller; from samtools mpileup-derived input, determine distributions of depth and strand-bias genome-wide, identify regions with abnormal depth of coverage, and estimate rates of indel error within repeat tracts. *Mutation Calling*: compare outgrowth and t0 counts to determine if sufficient evidence exists to call mutations, call genotypes, apply filters, and determine mutation identities. The “*run_pipeline”* command automatically performs all tasks following *Genome Preparation* (dark gray background)*.* *Depth Correction/Ploidy Detection*: following generation of processed alignments and coverage bedGraph files, muver commands can be utilized to generate distributions of observed allelic fractions and ratios of coverage to the mean as a function of distance from the chromosome end. Appropriate ploidy may be determined through plotting and visual inspection of the allelic fraction distribution. If bias is observed when plotting coverage ratios, parameters for correction may be determined via a non-linear least squares fit to a provided function, then passed to muver command “*correct_depths*”. Corrected depth distributions and filtered regions may then be determined from the resulting bedGraph files. Ploidy changes and any files resulting from the depth correction may be incorporated into the analysis through manual editing of the “Sample Info” table. This file may then be passed to the muver command “*call_mutations”* to generate updated output files.

**Supplemental Figure 2.**

Counts of observed alleles by allelic fraction. Allelic fractions below 0.1 and above 0.9 are omitted for clarity, else central peaks would appear insignificant compared to counts near allelic fractions of 0 and 1. Black diamonds indicate observed counts, solid lines are theoretical models for diploid (grey), triploid (red) and tetraploid (gold) cells, scaled to the area under the observed data. Examples are shown for (A) diploid, (B) triploid and (C) tetraploid yeast samples. (D-F) Where half of the population has a different chromosome count than the founder cell, the resulting theoretical allelic fraction curves are shown for a 2n to 3n expansion (“di to tri”; light green), a 3n to 4n expansion (“tri to tetra”; dark green), a 3n to 2n contraction (“tri to di”; blue), and a 4n to 3n contraction (“tetra to tri”; purple). (D) An example of a diploid-triploid mix that appears to have originated from a triploid founder. (E-F) The effects of library contamination on apparent allelic fraction (samples and contaminants are diploid).

**Supplemental Figure 3.**

Measures of read distribution differences between ancestor and descendant. Over 21,000 variants were called by GATK HaplotypeCaller in an unpublished *Saccharomyces cerevisiae* test set selected for high expected mutation counts and a complex spectrum of mutation types. One ancestral and four descendant samples were compared. Per-allele read counts were compared via binomial test for major alleles (top strand = *pT*, bottom strand = *pB*) or via chi-squared test for the distribution of reads across all alleles (*pχ*). Negative log_10_ of upper tail p-values are shown. The observed distribution of these values may be plotted as coordinates in three dimensions (transparent cubes). Variants were classified as positive mutations with stringent *pT* and *pB* cutoffs (blue "auto positive") and the remainder as manually assigned as either mutations (green "manual positive") or non-mutations (orange "manual negative"). (A-C) Plots are two-dimensional projections with the direction(s) of projection indicated on the adjacent small three-dimensional plot: the left plot shows the full range of p-values; the right plot shows a subset near the origin, corresponding to the red box on the left plot. (A) Left plots show the full range of p-values and right plots show a subset corresponding to the red box comparing (A) *pT* and *pB*, (B) *pT* and *pχ*, and (C) *pχ* versus the vector sum of *pT* and *pB* (*d*; i.e. the distance from the origin in panel A). Variants closer to an axis than the grey dashed line were rejected, including some manually assigned as mutations (black-bordered green "false negative"). The rest were accepted as mutations, including two that were manually called non-mutations (red circles). The circular arc portion of the dashed grey line is centered at the origin with a radius of -log_10_(*m*), where *m* is the p-value necessary to control family-wise error at 0.01 by the Šidák method.


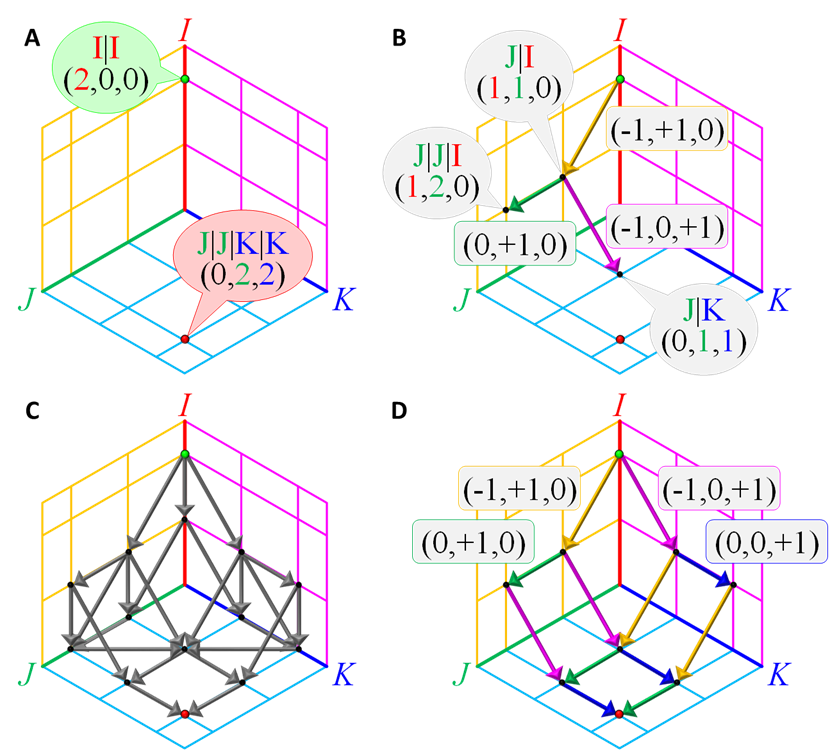


**Supplemental Figure 4.**

The pathway analysis determines the most parsimonious series of events (point mutations and/or copy number changes) that would explain the observed clonal genotypes in ancestral and descendant loci. (A) Observed ancestral (red dot) and descendant (bright green dot) genotypes are mapped onto an N-dimensional space where N is the number of observed alleles. (B) Changes parallel to an axis (green and blue arrows) represent copy number changes. Changes diagonal to axes represent point mutations (gold and magenta arrows). (C) All legal changes that reduce Euclidean distance are plotted. (D) Maximally parsimonious pathways are calculated and states shared by all are called as mutations. Where states exist in some but not all paths, mutations are called with ambiguities noted.


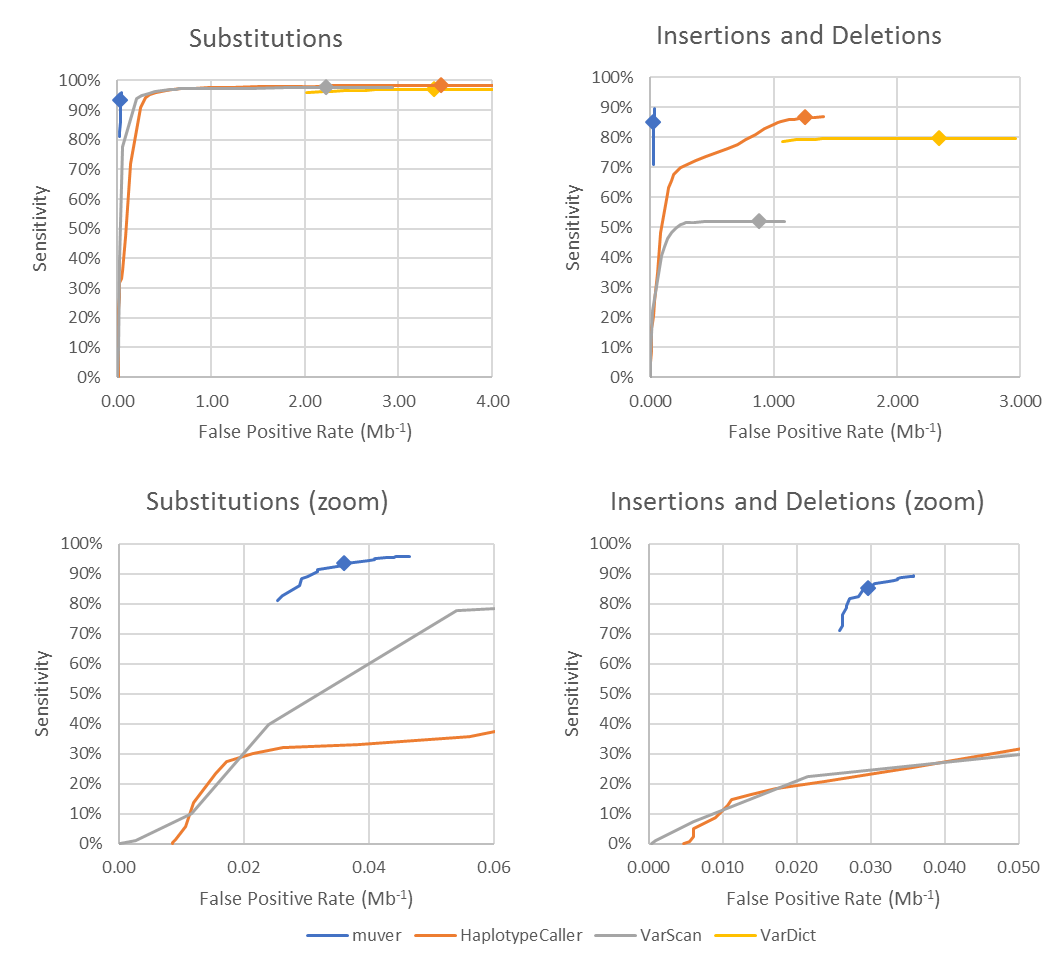


**Supplemental Figure 5.**

Sensitivity and false positive rates observed for muver (blue), VarScan (gray), HaplotypeCaller (orange), and VarDict (yellow). Diamonds indicate tool defaults for muver and VarScan, author recommended threshold of 0.05 for VarDict, and the commonly used calling threshold of 30 for HaplotypeCaller. Muver was assessed across a range of family-wise error rates from 2 x 10^-7^ to 0.9, VarScan for p-values from 1 x 10^-50^ to 0.1, HaplotypeCaller for calling quality thresholds of 10 to 10,000, and VarDict for p-values from 1 x 10^-6^ (the minimum non-zero value reported) to 0.1. See Supplemental Table 5-7 for values. Differences between the son and father of the Genome in a Bottle (GiaB) Consortium’s Ashkenazim Trio were detected based on a subset of the Illumina HiSeq 300x data set only. Rates were measured by comparison with GiaB high confidence variant calls. Due to filtering applied independent of the user-selected family-wise error rate, muver’s dynamic range is smaller than those observed for the other tools, however, none is capable of achieving such low false positive rates without a dramatic loss in sensitivity.

**
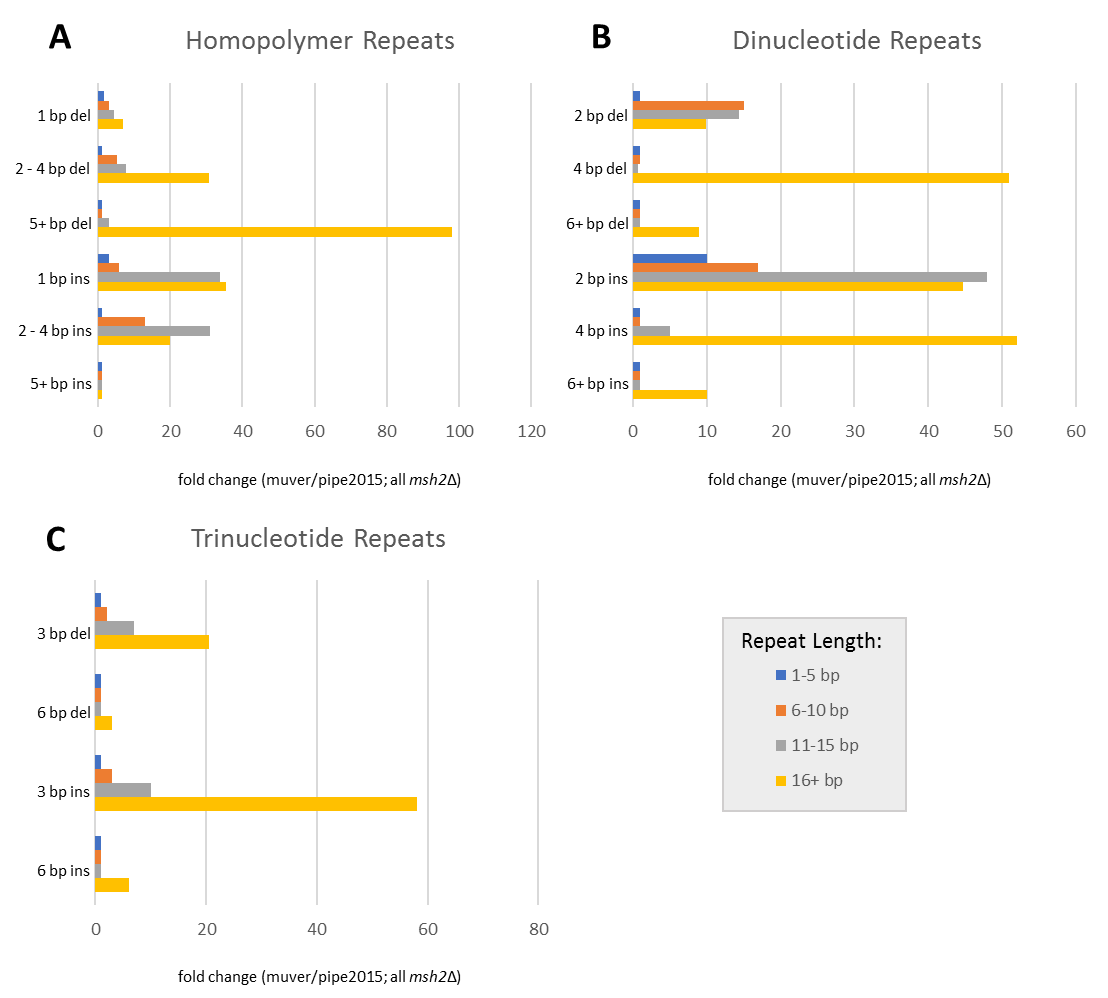
**

**Supplemental Figure 6.**

Fold change in mutation counts (muver over pipe2015) in repeat tracts of varying lengths for homopolymer repeats (A), dinuceotide repeats (B), and trinucleotide repeats (C), separated by type of event, and length of inserted or deleted sequence. These values are derived from all *msh2*Δ data sets examined. In calculating fold changes, pseudocounts were added to the numerator and denominator for categories where the pipe2015 totals were zero. Higher fold changes are associated with longer repeats and longer insertions or deletions, the most difficult contexts in which to identify mutations.


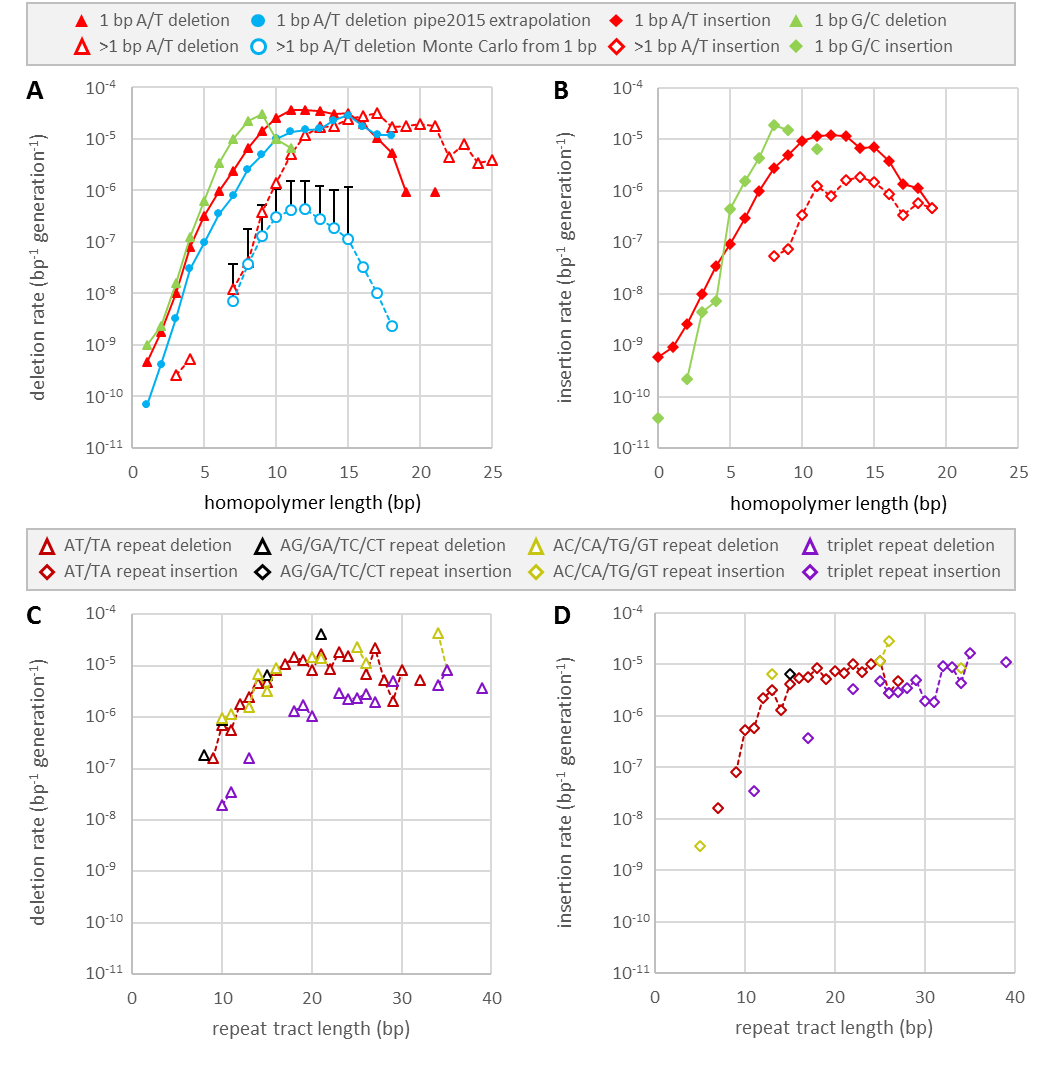


**Supplemental Figure 7.**

Muver-derived insertion and deletion rates in repeat tracts in *pol2-M645G msh2*Δ samples. Rates (per bp per generation) of single-base (filled) and multi-base (open) deletions (triangles) and insertions (diamonds) are shown versus tract length for repeats with unit length of 1 (homopolymers; A-B) or greater than 1 (dinucleotide and triplet repeats; C-D). Single-base deletion rates in A/T homopolymers (closed blue circles) were extrapolated statistically from previous results (9). Multi-base deletion rates in A/T homopolymers (open blue circles) were modelled assuming that they were generated by sequential single-base events (error bars represent upper 95% confidence limits from 5000 simulations).

**SUPPLEMENTAL RESULTS AND DISCUSSION**

**Subclonal mutation calling.**

Muver reports subclonal mutations but further reference-specific filtering is needed. Mutations that occur in the first few cell divisions after the final passage will be observed at subclonal frequencies. While rare, these mutations could provide mechanistic insights. Subclonal mutation rates should not vary over the course of a mutation accumulation experiment and an increase or decrease in subclonal mutation rate could indicate acquired mutator or mutation suppressor phenotypes. Subclonal rates that exceed clonal rates may indicate selection against new mutations. Such selection would mean that clonal rates are underestimates.

The inclusion of subclonal mutations in muver models improves the calling of clonal mutations by correcting for various errors and increases robustness versus contamination and other sample heterogeneity. Through observation of many data sets, we have found that even after extensive filtering, the rate of false positive subclonal calls is higher than desired. We suspect that the main reason for this is HaplotypeCaller’s method of read assignment, which reports counts based on a probabilistic alignment to the called haplotypes only, resulting in some scenarios in which reads that would normally be attributed to PCR error or mismapping are instead interpreted as an increase in frequency due to the presence of a subclonal allele. By tuning HaplotypeCaller’s parameters to increase its sensitivity, and induce a more diverse set of reported alleles, this affect has been mitigated. It is also mitigated by the inclusion of a large number of outgrowth samples, increasing the chances of a heterozygous genotype call including a lower frequency allele. Future development of muver will include an effort to improve the accuracy of input read counts through further modification of HaplotypeCaller’s parameters or it’s replacement with another method that incorporates local re-assembly of reads.

**Data set provenance and changes in usage.**

Muver results revealed unappreciated aspects of data sets analyzed with the previous pipeline (pipe2015; (8)). Five samples were considered for exclusion from further analysis: mutations that were previously missed but were called by muver revealed that one *wild type* sample (TAK114) was an outgrowth of another (TAK70) that had acquired a mild mutator phenotype; and muver allelic fraction graphs showed that two *msh*Δ samples (TAK262 and 266) and one *pol2-M644G msh2*Δ sample (TAK180) and one *pol1-L868M msh2*Δ sample (TAK199) had apparent mixed ploidy (examples in Supplemental Figure 2D-F). Further analysis revealed that the aberrant allelic fractions signaled more or less minor library contamination. Among those, only TAK114 and TAK199 were excluded. Also excluded was *pol1-L868M msh2*Δ sample TAK269 that was found to have a mean genome-wide sequencing depth of 19, falling below muver’s default threshold of 20.

The remaining three contaminated samples were retained, as their mutation spectra were essentially identical to sibling outgrowths (R^2^ >0.98) and their mutation rates fell within the ranges set by their siblings. In retrospect, the allelic fraction plots delivered by muver were diagnostic for the contamination level: for TAK180, the central diploid peak split into two centered at 0.42 and 0.58, or 0.5 ± 0.5 x the contamination fraction (~16% contamination measured at select loci; Supplemental Figure 2E). Since TAK262 and 266 were each contaminated by two independent sources, estimated at 12% and 18% for approximately 30% total contamination, the allelic fraction peaks are more complex (Supplemental Figure 2F). Nonetheless, in all three cases it appears that the bulk of reads attributable to contaminant alleles were interpreted by muver as subclonal mutations and called as such, leaving clean clonal mutation lists. This was surprising, as muver was not designed to handle heterogeneous samples. To assess the potential harm of such contamination, we performed an analysis similar to the previously discussed “virtual outgrowth”. In this case, reads derived from a *pol3*-L612M *msh2∆* sample were combined with reads derived from a *WT* sample at a rate of 10-50%. Mutations were then called using muver, with default parameters, against a separate set of reads derived from the same *WT* sample. At 10% contamination, no miscalls were observed, and at 20%, higher than the level of any single source described above, only 19 were observed (Supplemental Table 4). Compared to the potential maximum of 6,017, the count of mutations called when the full set of *pol3*-L612M *msh2∆* reads were compared to *WT*, this value is quite low, approximately 1.3%. Even at 50% contamination, less than 8.5% of the maximum were called, however, further study and refinement are needed to define limits of muver’s capabilities with heterogeneous samples like human tumor tissues.
